# Ecoregional and temporal dynamics of dugong habitat use in a complex coral reef lagoon ecosystem

**DOI:** 10.1038/s41598-021-04412-3

**Published:** 2022-01-11

**Authors:** Solène Derville, Christophe Cleguer, Claire Garrigue

**Affiliations:** 1 UMR ENTROPIE (IRD-Université de La Réunion-CNRS-Laboratoire d’excellence LabEx-CORAIL), 98800 Nouméa, New Caledonia; 2 Opération Cétacés, 98802 Nouméa, New Caledonia; 3 Marine Mammal Institute, Oregon State University, 2030 SE Marine Science Dr., Newport, OR 97365 USA; 4 Centre for Sustainable Aquatic Ecosystems, Harry Butler Institute, Murdoch University, Murdoch, WA 6150 Australia; 5 Centre for Tropical Water and Aquatic Ecosystem Research (TropWATER), James Cook University, Townsville, 4811 Australia

**Keywords:** Behavioural ecology, Conservation biology, Animal migration

## Abstract

Mobile marine species display complex and nonstationary habitat use patterns that require understanding to design effective management measures. In this study, the spatio-temporal habitat use dynamics of the vulnerable dugong (*Dugong dugon*) were modelled from 16 satellite-tagged individuals in the coral reef lagoonal ecosystems of New Caledonia, South Pacific. Dugong residence time was calculated along the interpolated tracks (9371 hourly positions) to estimate intensity of use in three contrasting ecoregions, previously identified through hierarchical clustering of lagoon topographic characteristics. Across ecoregions, differences were identified in dugong spatial intensity of use of shallow waters, deeper lagoon waters and the fore-reef shelf outside the barrier reef. Maps of dugong intensity of use were predicted from these ecological relationships and validated with spatial density estimates derived from aerial surveys conducted for population assessment. While high correlation was found between the two datasets, our study extended the spatial patterns of dugong distribution obtained from aerial surveys across the diel cycle, especially in shallow waters preferentially used by dugongs at night/dusk during high tide. This study has important implications for dugong conservation and illustrates the potential benefits of satellite tracking and dynamic habitat use modelling to inform spatial management of elusive and mobile marine mammals.

## Introduction

Understanding the drivers of spatio-temporal variability in habitat use by endangered species can inform conservation planning^1^. The marine environment is dynamic in nature and marine animals constantly respond and adapt to its changing physical and biological features^2^. Species-environment relationships are therefore inherently nonstationary, as they do not remain constant through space and time^3^. Indeed, habitat use may vary at multiple spatio-temporal scales^4^, displaying complex regional geographic adaptations or following stochastic (extreme events), cyclic (diel and tidal cycles) or continuous patterns (climate change^5^). Misunderstanding of these dynamic associations of species with their environment can impair current efforts to model^3,6^ and anticipate spatial conflicts between marine wildlife and anthropogenic activities (traffic, fishing, tourism, industries etc.). Dynamic and transferable approaches to study and model animal habitat use are therefore needed to avoid mismatches in the design of protective measures^7^.

Over the last decades, technological advances in the development of electronic tags for animal satellite tracking have revolutionized our capacity to monitor marine animal movements and address conservation issues^8,9^. Satellite telemetry benefited the study of highly mobile species such as marine mammals, allowing for the collection of individual behavioral data over relatively long time periods and in ecosystems otherwise difficult to study. For example, satellite telemetry has provided important insights into the dynamic space use patterns of marine mammals and their relationships to static environmental conditions (e.g., seabed topography). Telemetry revealed how marine mammals may respond to the fluctuations of coupled physical-biological conditions (e.g., ocean circulation and productivity) influencing the patchy distribution of their resources. In turn, this ecological knowledge may provide a robust basis for designing effective management tools or spatial protection to mitigate risks for endangered marine mammal species at local^10^ to global scales^11^.

Current research efforts to unravel the ecological drivers of dugong (*Dugong dugon*) distribution illustrate the added value of satellite telemetry to inform the conservation of this listed Vulnerable species^12^. Dugongs are broadly distributed in the Indo-Pacific coastal and island waters, where they occupy a diversity of habitats. Populations display localized high risks of extirpation in many parts of the dugong’s range outside Australia^13^. As a predominantly herbivorous marine mammal, the dugong is associated with seagrass meadows, hence playing a unique functional role in tropical and sub-tropical coastal ecosystems^14,15^. Indeed, dugong grazing influences the biomass, species composition and nutritional quality of seagrass beds^16–18^, as well as their resilience^19^ and dispersal^20^. Dugong regional distribution has mainly been documented through dedicated large scale aerial surveys in Mozambique, the Arabian Gulf, Australia, New Caledonia, and Malaysia^7,13,21–24^ or through fishermen questionnaires^25,26^. While the former is a lot more sophisticated and accurate both techniques are restricted in time (they represent snap-shots of daytime distribution) and analyzed at a coarse scale that limits the understanding of regional habitat use and spatio-temporal variations. Finer descriptions of dugong movements and habitat selection were made possible with satellite tracking undertaken over Australian continental shelf waters (e.g., Moreton Bay, Hervey Bay, Gulf of Carpentaria, West Kimberley^27–33^), as well as in Indonesia^34^ and New Caledonia^35^. These studies have suggested that dugong movements are heterogeneous^32^, but show regionally-adapted responses to seasonal, tidal and diel cycles. In some areas, dugongs venture to inshore areas more at night, often to feed over intertidal seagrass meadows at high tide^28,32,36,37^, presumably to avoid daytime human activities. Furthermore, there is some evidence of dugongs using tidal currents to save energy during their movements between key habitats^38^. Very few studies have attempted to compute the physical characteristics of the ecosystems in which dugongs are tracked to investigate how these characteristics vary across regions and how they may influence dugong movement and habitat use^30^. Such characteristics include, among others, depth, and distance to topographic features such as reef patches, barrier reefs or the coast.

New Caledonia is located near the eastern margin of dugong distribution in the Pacific^12^. Parts of the lagoons surrounding its main island are listed as a UNESCO World Heritage Site^39^. They are ringed by one of the world’s longest barrier reefs and support some of the most diverse coral reef ecosystems^40^. The dugong is an emblematic species of the island, represented by a fragile^41,42^, yet globally important population^43,44^. Extensive aerial surveys have provided a general overview of its distribution, with the highest densities being observed on the western coast^7^. Moreover, satellite tracking conducted in this area has revealed a great heterogeneity of movements across individuals and identified multiple core areas of use^35^. Dugongs were found to use diverse habitats, from intertidal coastal seagrass meadows to the deeper waters of the fore-reef shelf outside the barrier reef. However, past studies have not modeled the spatio-temporal variations of these complex space use patterns in New Caledonia. For instance, from the dugong tracking studies conducted in Australia, the tidal cycle could be hypothesized to determine the accessibility to seagrass meadows in the shallowest parts of the lagoon^36^. Moreover, marine traffic, recreational activities and fishing occurring during the daytime in the most populated parts of the lagoon^45^ may result in a diel pattern of habitat use by dugongs. Understanding and incorporating these fine-scale dynamic patterns into models of dugong habitat use may help refine spatio-temporal management measures.

Based on a compilation of available and newly acquired satellite tracking data along the western coast of New Caledonia, this study aims to understand ecoregional variability in the spatio-temporal dynamics of dugong habitat use in a coral reef lagoon ecosystem. Geographic variability in habitat use was assessed by modelling dugong residence time relative to lagoon topography and seagrass meadows within three contrasting ecoregions. Temporal variability in habitat use was assessed with respect to the tidal and diel cycles hypothesized to affect dugongs through relationships to seagrass accessibility and to disturbances such as human activities. Predicted patterns of habitat use were cross-validated with density maps generated through aerial surveys^7^ to evaluate the complementarity of tracking data to identify key dugong habitats.

## Results

**Figure 1:**
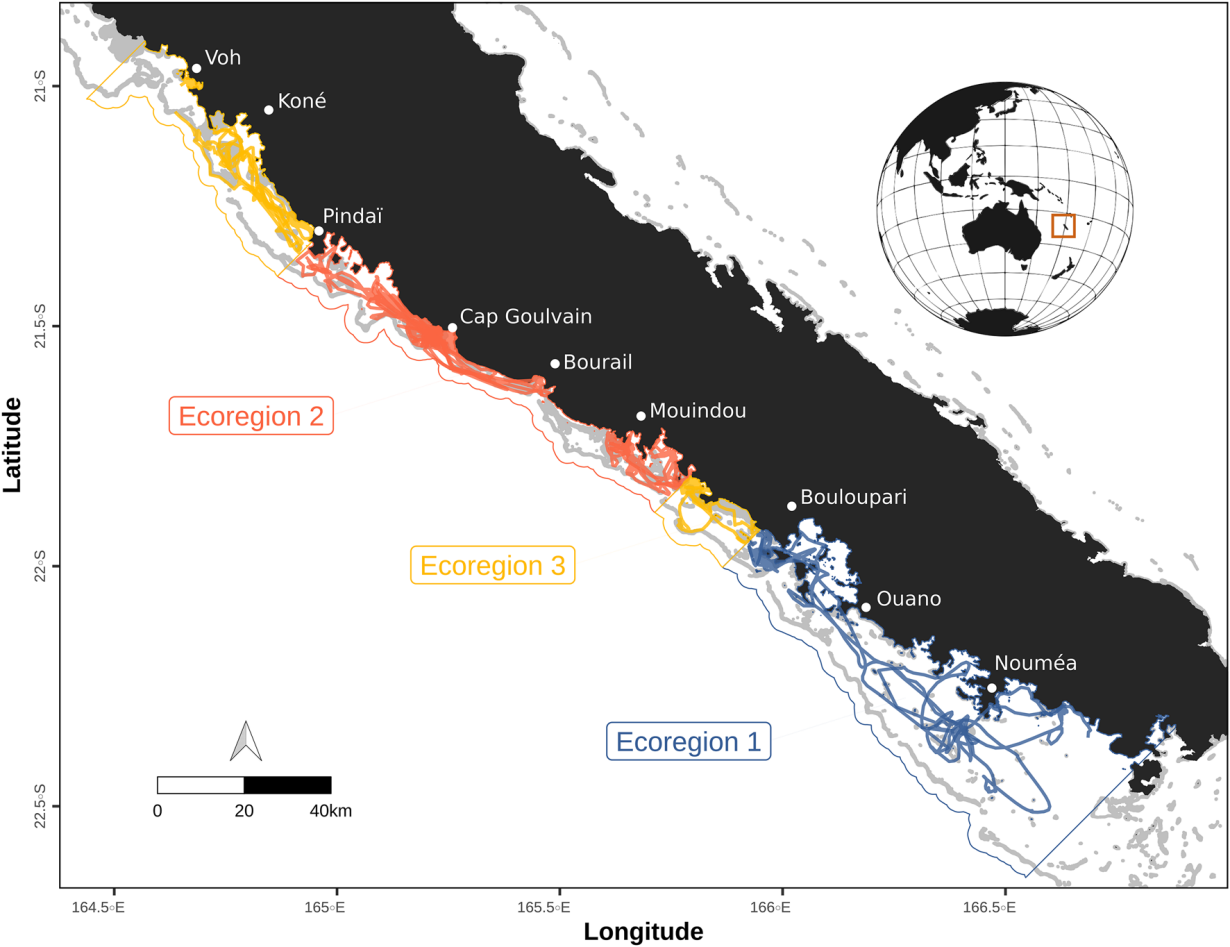
Interpolated dugong GPS tracks recorded in 2012, 2013 and 2019 over the west coast of New Caledonia, South Pacific. The 16 tracks are split in different colors depending on the ecoregion they are in. Ecoregions identified through hierarchical clustering of environmental lagoon characteristics are represented with colored polygons. Ecoregion 3 is divided into two separate parts, one to the north of ecoregion 2 and another in between ecoregion 1 and 2. Land is shown in black and shallow reefs are shown in grey (shapefile source: Millenium Coral Reef Mapping Project^69,70^). The map was created with R (version 3.6.3, www.r-project.org^86^).

Three ecoregions were identified over the western lagoon of New Caledonia (Fig. 1, Supplementary Fig. S1, Table 1). Ecoregion 1 covered the southern part of the study area and was characterized by relatively deep waters included within a wide lagoon, more than twice as large as in the rest of the study area. The lagoon in ecoregion 1 is also relatively more open to oceanic waters than the other ecoregions are. Ecoregion 2 was the shallowest. Its lagoon was particularly narrow and did not include intermediate reef patches between the barrier reef and the fringing reef; consequently, it was characterized by a greater distance to intermediate reefs than the other ecoregions. This region was also characterized by steep slopes inside the channels. Finally, ecoregion 3 was composed of two separate lagoon sections, one to the north of the study area, and the other located between ecoregions 1 and 2. Ecoregion 3 was characterized by shallow waters, but more variable in depth, with a medium lagoon width scattered with islets and intermediate reef patches.

**Table 1:** Characteristics of the ecoregions (mean ± S.E.M over n slices or over n cells) identified along the New Caledonia western coast lagoon.

	Ecoregion 1	Ecoregion 2	Ecoregion 3
n_slices	5	5	4
Surface area (km^2^)	443.3 ± 84.7	132.5 ± 23.6	197.3 ± 4.8
Sum of channel widths (km)	10.1	7.0	7.0
n_cells	8876	2659	3170
Depth (m)	16.9 ± 0.12	6.5 ± 0.30	7.1 ± 0.21
Slope (°)	0.7 ± 0.02	1.2 ± 0.07	0.9 ± 0.05
Distance to intermediate reef patches (km)	3.5 ± 0.03	25.8 ± 0.26	3.6 ± 0.04

The sixteen adult dugongs were tracked from 1.7 to 173.4 days (mean = 27.0 days ± S.E.M 10.7, Table 2), allowing for the collection of 6806 filtered GPS locations, resulting in 9371 hourly locations after interpolation (Fig. 1). Swim speed and residence time were estimated for 9047 of these locations (Table 1). Mean swim speed per individual varied from 0.3 km/h (± S.E.M 0.008, individual J) to 1.2 km/h (± S.E.M = 0.06, individual F), with a maximum observed swim speed of 3.9 km/h (individual D).

**Table 2:** Summary of the 16 interpolated dugong GPS tracks recorded in 2012, 2013 and 2019 over the west coast of New Caledonia, South Pacific.

ID	Tag and release time	Tagging position			Sex	Duration (days)	# Locations per ecoregion			Swim speed (Mean ± S.E.M)	Residence time (Mean ± S.E.M)
		Ecoregion	Latitude	Longitude			1	2	3		
A	02/03/2012 14:00:00	3	− 21.82363	165.79988	M	24.4		296	291	0.6 ± 0.03	10.4 ± 0.3
B	03/03/2012 16:00:00	3	− 21.783614	165.65758	F	20.2	37	309	140	0.7 ± 0.03	9.3 ± 0.4
C	24/09/2013 14:36:00	1	− 22.289016	166.46700	M	1.7	42			0.9 ± 0.09	3.9 ± 0.5
D	27/09/2013 10:00:00	1	− 22.3166	166.36666	F	12.5	259			0.9 ± 0.05	5.3 ± 0.3
E	28/09/2013 11:28:00	1	− 22.327116	166.37951	F	38.5	856		4	0.6 ± 0.03	18.4 ± 1.0
F	01/10/2013 09:02:00	1	− 21.51646	165.19702	F	10.0		179	31	1.2 ± 0.06	3.5 ± 0.2
G	02/10/2013 08:57:23	2	− 21.5101	165.17909	F	2.2		55		1.1 ± 0.14	3.4 ± 0.2
H	02/10/2013 09:18:31	2	− 21.5089	165.17313	F	18.7		408		0.9 ± 0.03	4.2 ± 0.1
I	03/10/2013 08:08:30	2	− 21.52041	165.21535	M	12.4		203	62	0.5 ± 0.04	15.7 ± 0.7
J	03/10/2013 08:41:51	2	− 21.51386	165.20517	M	73.4		1542		0.3 ± 0.008	40.6 ± 0.9
K	04/10/2013 08:18:20	2	− 21.52844	165.21561	M	173.4		3594		0.4 ± 0.006	16.7 ± 0.3
L	03/10/2013 09:41:44	2	− 21.52932	165.19276	F	4.3		86		1.2 ± 0.14	5.9 ± 0.7
M	04/10/2019 09:20:00	3	− 20.99966	164.65275	F	3.3			81	0.6 ± 0.06	8.2 ± 3.5
N	10/10/2019 10:28:00	3	− 21.14301	164.72916	F	6.6		8	151	0.9 ± 0.07	8.6 ± 8.0
O	11/10/2019 11:45:00	3	− 21.15866	164.74955	F	6.8			164	0.5 ± 0.04	8.8 ± 5.3
P	11/10/2019 13:29:00	3	− 21.160833	164.74356	F	23.8			573	0.4 ± 0.01	22.8 ± 21.1

ID = individual dugong code, Tag and release time = in local time zone, Tagging position = ecoregion and position in coordinate system EPSG 4326 (latitude and longitude in decimal degrees) where animal was tagged, Sex = M for males and F for females, Duration = duration of the interpolated track in days, # Locations per ecoregion = number of interpolated locations per ecoregion per track, Mean swim speed (km/h) and mean residence time (hrs). Individual tracks are provided in Supplementary Figure S2.

**Figure 2:**
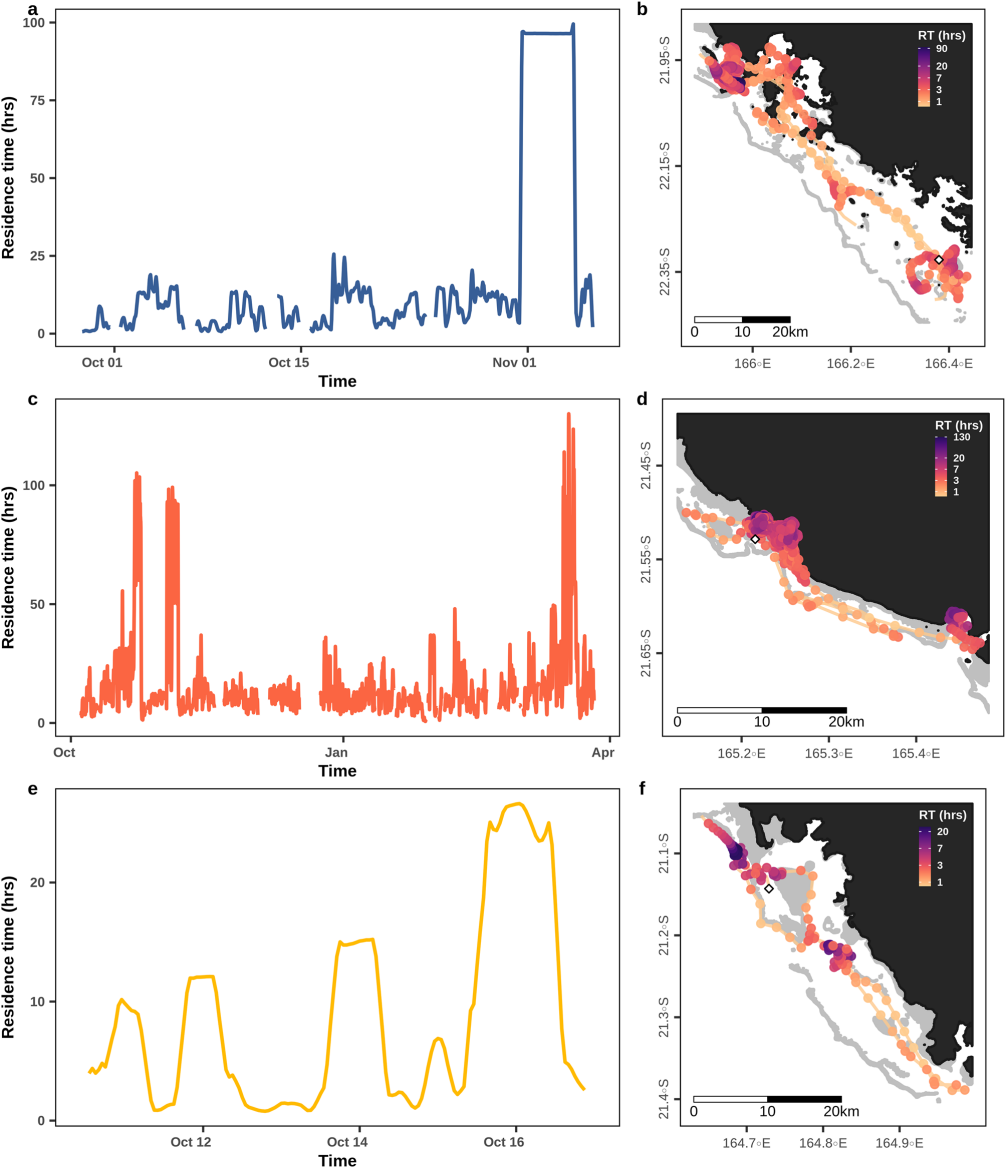
Residence time calculated over the interpolated GPS tracks of three different dugongs distributed in each of the New Caledonia west coast ecoregions. Panels (**A**,**B**): individual E in ecoregion 1. Panels (**C**,**D**): individual K in ecoregion 2. Panels (**E**,**F**): individual N in ecoregion 3. In the panels (**B**,**D**, and **F**) the color gradient representing residence time at each location is log scaled. The locations of tag deployments are shown with white diamond shapes. Land is represented in black and shallow reefs in grey (shapefile source: Millenium Coral Reef Mapping Project^69,70^). The maps were created with R (version 3.6.3, www.r-project.org^86^).

Residence time varied both among individuals and within a given track as dugongs switched between transit-like and Areas of Restricted Search (ARS) movements (Fig. 2, and Supplementary Fig. S2 for all individuals). Males showed significantly higher residence time (n = 5862, mean Residence Time (RT) = 22.1 h ± S.E.M 0.3) than females (n = 3185, mean RT = 12.5 h ± S.E.M 0.3), meaning that they had slower and/or more sinuous paths on average (linear mixed model: χ^2^ = 4.4, *p* = 0.036*). Residence time was not significantly different across ecoregions (linear mixed model χ^2^ = 0.5, *p* = 0.796).

Almost all locations were inside the lagoon (99.3%), although some individuals ventured outside the barrier reef: one in ecoregion 3 (individual N, two excursions), five in ecoregion 2 (individuals B, G, J, K, and L, eight excursions in total), and none in ecoregion 1 (Supplementary Table S3). These excursions lasted 8.1 h on average (± 0.48 S.E.M) with a maximum of 19 h spent outside the lagoon for individual G in ecoregion 2. Residence time during these excursions was generally low (mean = 3.6 h ± 0.55 S.E.M), indicating transiting behavior outside the barrier reef. An exception was found in ecoregion 3, where individual N swam outside the lagoon with mean residence time reaching 19.0 h (± 0.27 S.E.M) indicating ARS behavior on the outer slope of the barrier reef (Fig. 2F).

### Lagoon habitat use

**Figure 3:**
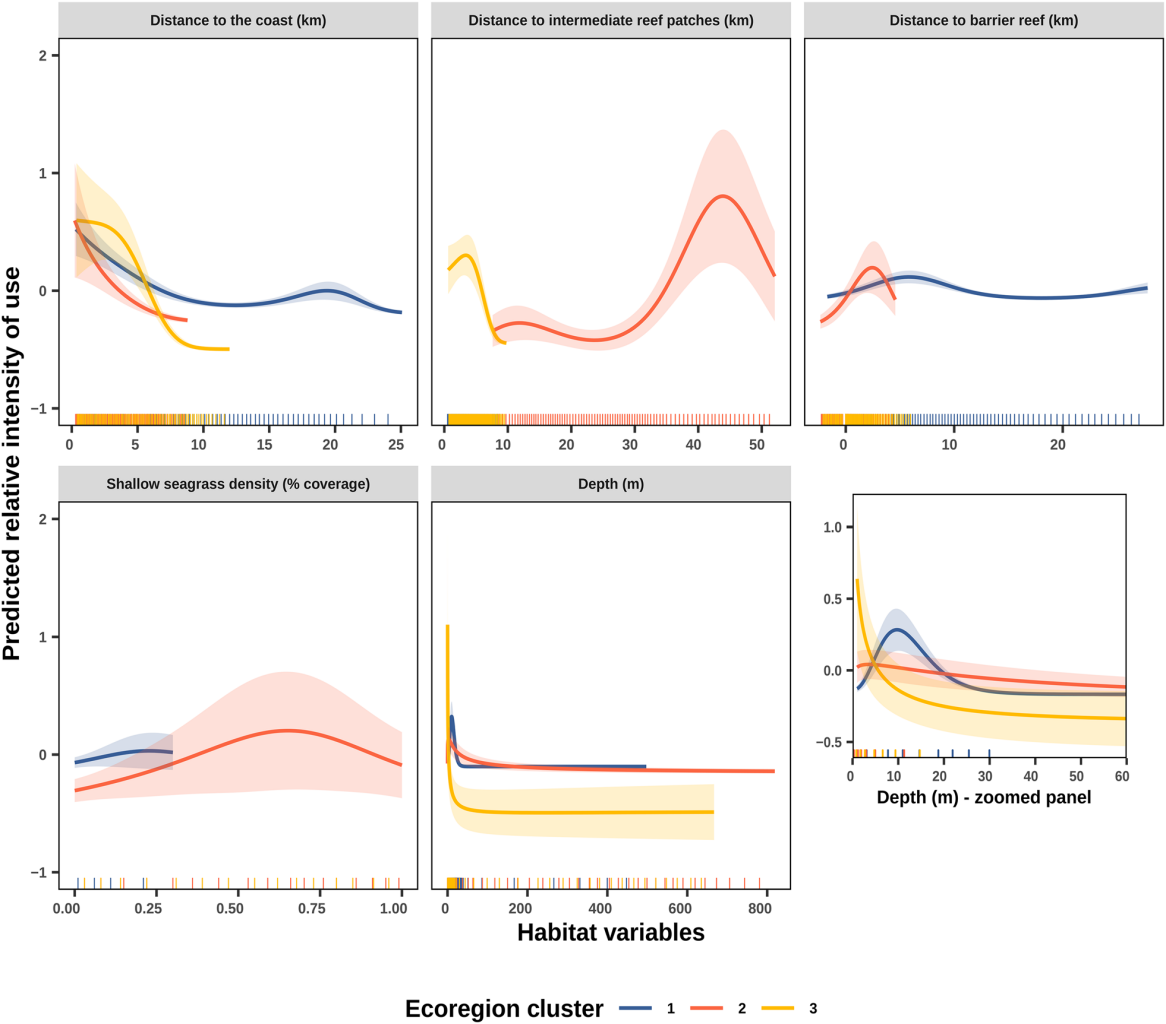
Partial response plots from a Generalized Additive Model between dugong habitat intensity of use and five environmental predictors (and a zoomed panel on response to depth in shallow-medium waters). The y-axis indicates the effect of the smooth function of each predictor upon the trend in dugong intensity of use, with higher values indicating increased intensity of use and potential for Area Restricted Search (predicted values were normalized to be centered on zero). Ecoregional smooth estimates are shown with different colors. Solid lines represent the marginal effect of each significant variable (with *p* value < 0.05) relative to dugong intensity of use. Shaded areas represent approximate 95% confidence intervals. Rug plots show the distribution of values per ecoregion for each predictor, with the 5% most extreme values being cropped out of the x-axis.

The model of dugong habitat use in the western coast lagoon of New Caledonia explained 43.3% of the deviance in the intensity of use observed over a 500 m resolution grid of 18,054 cells (Table 3). Dugongs showed a generally marked preference for shallow (< 20 m) and coastal waters inside the lagoon (< 5 km from the coast, Fig. 3). Their habitat use patterns varied across ecoregions, in relation to reefs and seagrass patches.

**Table 3:** Summary of the GAM habitat model selected to describe dugong intensity of use as a function of five environmental predictors per ecoregion.

Ecoregion	n	Discoast			Disreef			Disbar			Seagrass			Depth		
		χ^2^	edf	*p*	χ^2^	edf	*p*	χ^2^	edf	*p*	χ^2^	edf	*p*	χ^2^	edf	*p*
1	4	231	4.0	**< 0.001**	0.8	0.5	0.201	114	3.0	**< 0.001**	187	3.9	**< 0.001**	54	3.0	**< 0.001**
2	10	50	3.2	**< 0.001**	53	2.9	**< 0.001**	37	2.0	**< 0.001**	50	1.6	**< 0.001**	13	1.8	**< 0.001**
3	9	22	2.1	**< 0.001**	38	1.9	**< 0.001**	1	0.6	0.136	83	2.0	**< 0.001**	4	1.5	0.054

Significant relationships (*p* value *p* < 0.05*) are shown in bold. The number of individual dugongs contributing to the ecoregional models is reported by ecoregion (n). Edf = estimated degrees of freedom. DISCOAST = distance to the coast, DISTREEF = distance to intermediate reef patches, DISBAR = distance to the barrier reef, SEAGRASS = shallow seagrass (< 5 m) density, DEPTH = depth.

In ecoregion 1, dugongs occupied waters that were deeper than in the other two regions, and further away from the coast, but without reaching the immediate proximity of the barrier reef (Fig. 3). Moreover, the coverage in shallow seagrass patches was a significant predictor of dugong intensity of use in this region, although this relationship was weak.

In ecoregion 2, dugongs were constrained between the coastline and the barrier reef, spending time in proximity to both. Furthermore, shallow seagrass coverage was positively related to greater intensity of use. Finally, as the lagoon in this ecoregion is very narrow and does not include any intermediate reefs (only barrier and fringing), models predicted high intensity of use at a relatively great distance from intermediate reef (~ 45 km).

In ecoregion 3, high intensity of use was observed both inside and outside the lagoon despite the lagoon being relatively larger than in ecoregion 2 (Fig. 2F). This pattern was reflected in the absence of a significant effect on dugong intensity of use of distance to the barrier reef (Fig. 3). The coverage in shallow seagrass patches did not significantly influence dugong residence time. Nonetheless, dugongs were found to spend time close to intermediate reef patches and in shallow waters (< 2 m).

### Model cross-validation

Overall, the predictions of the model of dugong habitat use developed from the tracking data were positively correlated with the relative density estimates of dugongs generated from aerial survey data (Pearson r = 0.17, t = 21.7, *p* < 0.001); and the model helped to identify 4.3% of newly predicted habitats in the whole study area (and omitted only 0.01% of habitats). Positive correlation between the predicted intensity of use and the relative density estimates was particularly evident in the deep (10–60 m, Pearson r = 0.29, t = 25.85, *p* < 0.001) and medium depth waters (2–10 m, Pearson r = 0.16, t = 10.4, *p* < 0.001). The discrepancy between the two models in these depth ranges was minimal as less than 0.7% of grid cells were newly predicted as suitable habitat or omitted in deep waters. In medium depth waters, no grid cell was omitted and 3.0% highlighted newly predicted habitat. Very deep waters (> 60 m) had the lowest predicted intensity of use (mean = 0.03 h/km^2^ ± S.E.M 0.002) and included no newly predicted habitat.

**Figure 4:**
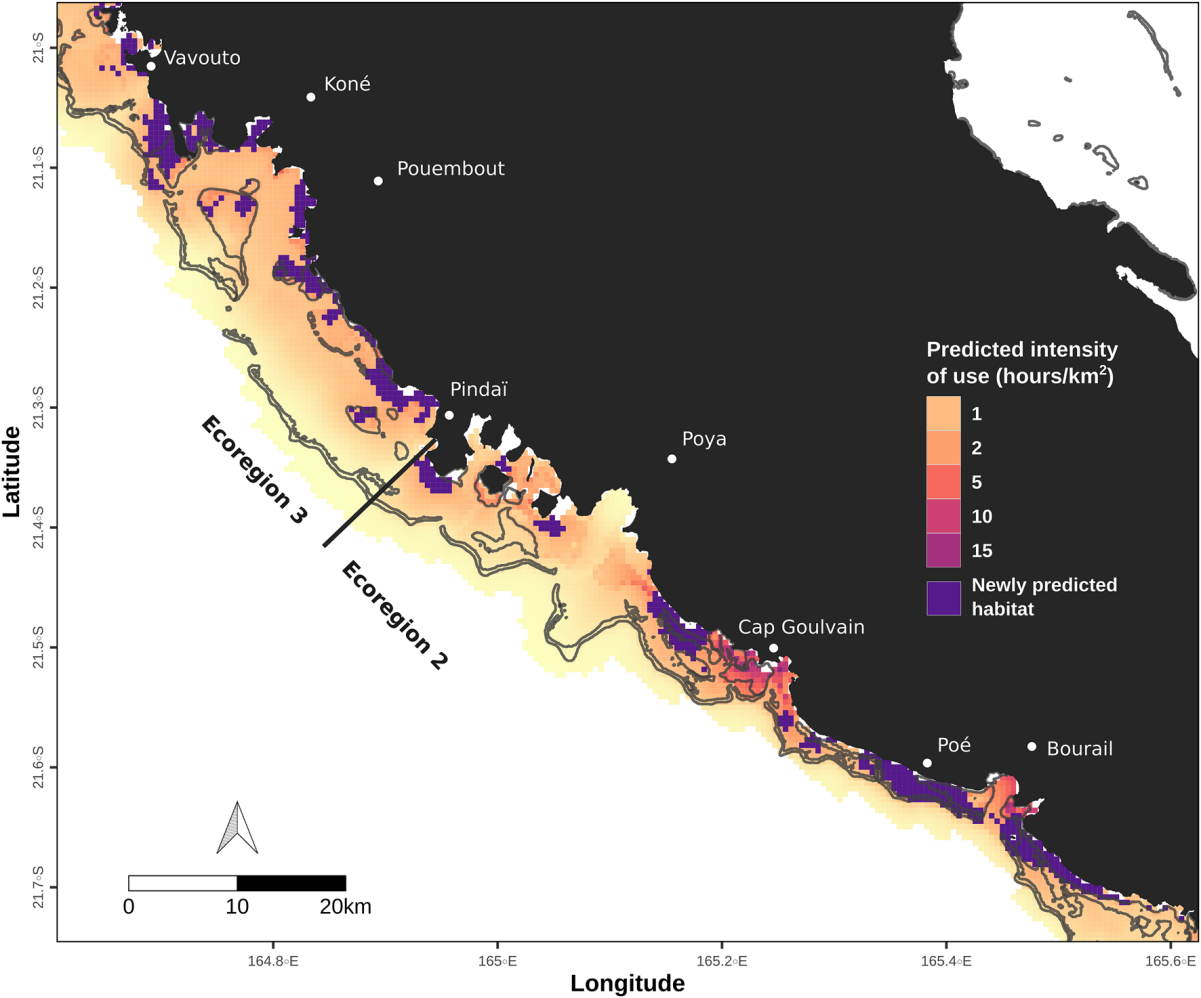
Map of predicted intensity of use zoomed over the northern border between ecoregions 2 and 3, overlaid with newly predicted habitats as revealed by cross-validation. Predicted intensity of use (h/km^2^) is shown on a colored scale. Newly predicted habitats (i.e. areas predicted with highly suitable habitat but where density from aerial surveys was classified as low) are shown in purple. Land is represented in black and shallow reef contours in grey (shapefile source: Millenium Coral Reef Mapping Project^69,70^). The map was created with R (version 3.6.3, www.r-project.org^86^).

In contrast, in shallow waters (0–2 m) the predicted intensity of use by dugongs was weakly correlated with relative dugong density estimates (Pearson r = 0.04, t = 2.1, *p* = 0.037). In this depth range, a relatively large proportion of the grid cells were classified as newly predicted (19.1%). Mapped discrepancies between predicted intensity of use and relative density estimates revealed that newly predicted habitats were primarily concentrated in shallow waters near the shoreline of ecoregions 2 and 3 (Fig. 4).

### Tidal and diel cycles

The magnitude and variability of the tidal cycle across the study period (2012, 2013–2014 and 2019) in New Caledonia is low. Sea surface heights reached 1.80–1.91 m during high tide, and were comprised between 1.10 and 1.28 m during low tide. The linear mixed models revealed that tidal and diel phase had a minor influence on the tracked dugongs’ residence time, with marginal R^2^ below 5% in any of the ecoregions (Table 4). In ecoregion 1, tidal and diel phase affected dugong movement when considered interactively (χ^2^ = 12.8, *p* = 0.005), with residence time on average greatest at low tide during the night (mean = 22.2 h ± S.E.M 4.7) and lowest at high tide at night (mean = 6.3 h ± S.E.M 0.7). In ecoregion 2, tidal phase had a significant overall influence on residence time (χ^2^ = 4.0, *p* = 0.044), in addition to affecting residence time in interaction with diel cycle (χ^2^ = 10.4, *p* = 0.016). In ecoregion 2, residence time was the greatest at low tide (mean = 21.8 h ± S.E.M 1.0). Finally, tidal and diel phases were not found to significantly affect residence time in ecoregion 3.

**Table 4:** Summary of the linear mixed model relating residence time or used depth range with tidal (high/low) and diel phase (dawn/day/dusk/night).

Response	Ecoregion	n_ind	n_loc	Tidal		Diel		Tidal x diel		Marginal R^2^ (%)	Conditional R^2^ (%)
				χ^2^	*p*	χ^2^	*p*	χ^2^	*p*		
Residence time	1	4	402	2.5	0.113	5.4	0.144	12.8	**0.005**	3.5	10.5
	2	9	1736	4.0	**0.044**	1.5	0.693	10.4	**0.016**	0.8	47.8
	3	8	490	3.2	0.072	3.8	0.278	7.3	0.06	2.6	16.9
Depth range	1	4	422	0.1	0.737	37.6	** < 0.001**	10.9	**0.012**	12.5	28.9
	2	9	1788	19.9	** < 0.001**	67.9	** < 0.001**	0.4	0.940	5.8	31.1
	3	8	510	4.6	**0.032**	32.3	** < 0.001**	31.4	** < 0.001**	6.4	37.3

The number of individual dugongs and locations contributing is reported by ecoregion (n_ind and n_loc respectively). Type III Wald χ^2^ tests are computed for each model (χ^2^ statistic and *p* value reported). Significant relationships (*p* value < 0.05*) are shown in bold.

**Figure 5:**
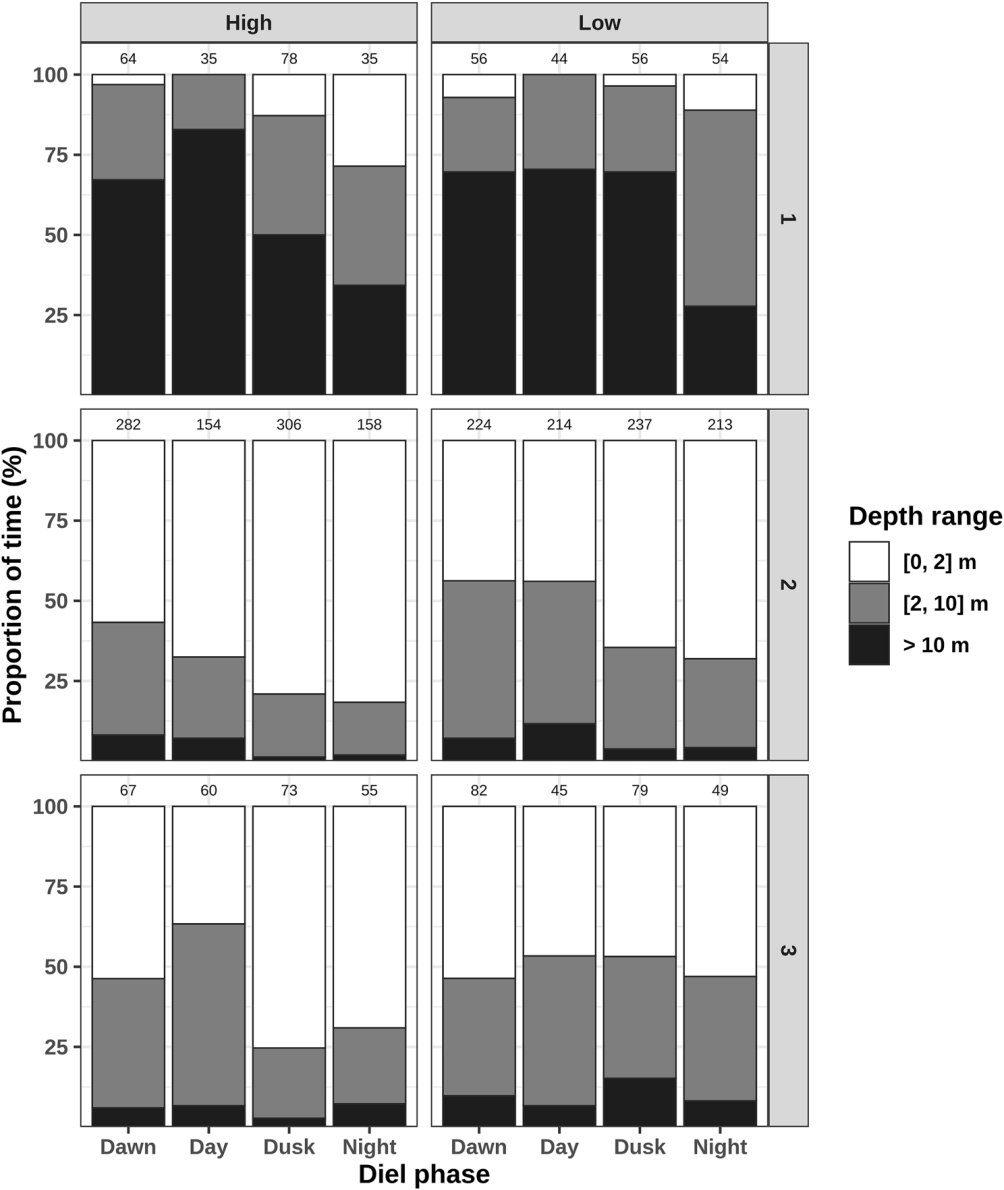
Effect of the tidal (High vs. Low tide phases) and diel cycles (Dawn, Day, Dusk and Night diel phases) on the depth range used by 16 dugongs tagged in 3 ecoregions (numbered 1–3 from top to bottom panels) over the west coast of New Caledonia, South Pacific. Seabed depth was extracted at hourly locations and binned into three categories fitting to the lagoon topography: shallow [0, 2] m, medium [2, 10] m, and deep > 10 m. Sample size in each ecoregion, diel phase and tidal phase combination are reported at the top of each of the bars.

Dugongs were found to occupy different depth ranges depending on tidal and diel phase (Table 4). They were found in shallower waters at dusk and night compared to day and dawn, and this effect was particularly obvious at high tide (Fig. 5). This pattern was the strongest in ecoregion 1, where the marginal R^2^ of the linear mixed model reached 12.5%. At high tide in this ecoregion, dugongs were located in shallow waters ([0, 2] m) 2.9% of the time during the day versus 51.4% of the time during the night (post-hoc Tukey test: df = 427, t-ratio = 5.5, *p* < 0.001).

Among the 10 excursions recorded outside the lagoon, the timing of excursions outside the barrier reef was significantly influenced by the diel cycle (χ^2^ = 8.4, *p* = 0.04), with dugongs mostly moving outwards at dawn (n = 6 out of 10, Supplementary Table S3). Dugongs swam back inside the lagoon more frequently at dusk or night (n = 6), but this pattern was not statistically significant (χ^2^ = 4.4, *p* = 0.2). Among these few excursions, dugongs were found to move in and out of the lagoon at any tidal phase (outwards movements: n = 4 at high tide, inwards movements: n = 3 at low tide). They also moved pass the barrier both during ebb (outwards movements: n = 3, inwards movements: n = 3) and flood (outwards movements: n = 2, inwards movements: n = 3).

## Discussion

Movement and habitat use studies enhance the ecological understanding of wild animals and can enhance management initiatives^8,46,47^. In this study, we demonstrate the importance of dynamically modeling the associations of species with their environment based on satellite tracking data. To that extent, the New Caledonian dugong population is a particularly interesting case study because of the great diversity of habitats and conditions encountered by the species within the coral reef lagoons^40^. The discovery of geographic and temporal variations in dugong space use complements prior distribution knowledge acquired from aerial surveys to support local management of this species.

This study was based upon a sample size of 16 tracked animals. While this is a small sample when compared to the size of the dugong population of New Caledonia (estimate relative abundance range between 426 ± 134 and 717 ± 171 individuals between 2008 and 2012^44,48^) our analysis yielded a robust habitat use model (43.3% deviance explained). Model cross-validation with aerial survey data suggested that key dugong habitats have been comprehensively delineated by the tracking data and modelling approach, within the regions where dugongs were tracked^49^, thereby adding to the representability of the tracking data. The predicted intensity of use by dugongs was highly correlated with dugong densities estimated from the time series of aerial surveys that were conducted around the main island of New Caledonia for the purpose of population assessments^7^. The habitat use model detected almost all of the preferred areas identified during the aerial survey (99.99% of grid cells). Such integration or cross-validation of data collected from complementary platforms of observations (aerial surveys, boat-based surveys, telemetry or opportunistic sightings) is increasingly applied to enhance the performance of animal distribution and habitat use models (e.g.^50–53^). Here, we showed the added value provided by satellite tracking, to complement dugong distribution and habitat knowledge acquired from aerial surveys designed for population assessment. The former provides temporally continuous data only over a limited number of individuals, while the latter provides population level data but only at a snap-shot in time. Model cross-validation pointed to the temporal bias of aerial surveys, which had failed to identify some key dugong habitats. Indeed, the importance of very shallow waters was underestimated by aerial surveys that could not detect their intense nighttime use by dugongs. In conclusion, even a relatively small number of satellite tags is useful to validate and complement coarse scale dugong distribution data provided by aerial surveys, hence filling ecological knowledge gaps.

A standardized approach using lagoon topographic variables was developed to identify key ecoregions across the western coast of New Caledonia where dugongs were tracked. We identified three ecoregions with distinct physical characteristics each of which, when combined with environmental and temporal variables, differentially influenced the habitat use patterns of the tracked individuals. While this spatial blocking approach inevitably divided the sample into smaller sets (with ecoregion 1 and 3 being relatively less sampled, Table 2), it provides a finer understanding of region-specific ecological relationships^54,55^. Indeed, the geography and topography of the identified ecoregions may have directly or indirectly influenced dugong habitat use. For instance, predation risk varies spatially, hence probably influencing dugong habitat use differentially across ecoregions. Like in Shark Bay, Australia^15,16,56^ tiger sharks *(Galeocerdo cuvier)* appear to prey upon dugongs in New Caledonia^35,43^, but little is known about the magnitude and the spatio-temporal distribution of this driver here. In addition, ecoregions clearly differed by the nature and intensity of anthropogenic activities that they encompass, which may explain some of the variability in the habitat use by dugongs. Ecoregion 1 is the urban center of New Caledonia, where roughly 70% of the human population lives (2019 census, www.isee.nc). In this region, the marine traffic and recreational use of the lagoon mainly occur during the day in proximity to the coast, and to islets and intermediate reef patches, and are by far the greatest in the main island^45,57^. These activities may be a cause of disturbance to dugong’s feeding activities and contribute to their diel pattern in habitat use, as hypothesized for dugong tracked at Burrums Heads in Eastern Queensland, Australia^36,58^. Indeed, vessel traffic in shallow waters has been recognized to trigger rapid flight response from dugongs that would generally attempt to escape in the deeper waters^31,59,60^. It is also a well know source of disturbance to the Florida manatees (*Trichechus manatus latirostris)*^61,62^. This avoidance could explain why the dugongs tracked in Ecoregion 1 barely used shallow waters during the daytime, while it comprised more than half of their time during the night. As nautical activities are increasing in Ecoregion 1, our findings could help local managers develop adequate management plans to ensure sustainable coexistence between dugongs and vessels.

Observed ecoregional differences in dugongs’ response to seagrass coverage and seabed depth may be better understood in light of known differences in species composition and distribution of seagrass meadows along the western coast of New Caledonia. In ecoregion 1, the present model supports prior utilization distribution analyses^35^, which show that the dugongs tracked in this region remained distant from both the inshore areas and the barrier reef, and primarily focused on the middle of the lagoon. There, seagrass meadows occur at the bottom of the intermediate reefs and islets slopes, in depths greater than 5 m. Indeed, the extent of ecoregion 1 roughly matches with the southwestern lagoon of New Caledonia that has been subject to a thorough inventory of macrophyte biodiversity, biomass and distribution^63^. Among the macrophyte associations identified through this study, mono-specific seagrass meadows of *Halophila decipiens* (possibly including *H. capricorni*^64^) were found in mean depths > 20 m, and covered up to a quarter of the area. Multi-specific meadows (*Cymodocea serrulata, Halodule uninervis, Syringodium isoetifolium*) growing at a mean depth of 11 m were also well represented (10% of the area). Although of major ecological importance, such seagrass meadows > 5 m deep are not represented in the compiled maps of shallow seagrass coverage used in the present model of dugong habitat selection^64^. The weak but positive relationship found between dugong intensity of use and seagrass coverage in ecoregion 1 might be strengthened by further mapping of the distribution and composition of deep seagrass meadows. This pattern might also occur in ecoregion 3 where dugongs were not detected to spend a significant amount of time in areas with dense seagrass coverage. Although the multiple other environmental variables included in the model have proved sufficient to produce relatively robust predictions of dugong key habitats, the description of underlying ecological relationships would benefit from improved seagrass mapping, specifically deep seagrass communities. Conversely, finer knowledge of New Caledonian dugong diet and resource selection patterns would contribute to direct research efforts towards seagrass communities of highest interest.

When the tide permitted, and particularly at night, dugongs made significantly higher use of shallow waters known to be covered with seagrass meadows, specifically over the intermediate reef plateaus surrounding islets in ecoregion 1 and the fringing reefs and inner bays of ecoregion 2. While tracking durations were relatively short on average (Table 2), they were distributed in such way that the effect of tidal and diel cycles could be investigated independently. These results corroborate the tidal and diel patterns in dugong-seagrass associations observed in other regions in Australia such as Hervey Bay, eastern Australia^37^ and Beagle Bay, northwestern Australia^28^. Dugongs in Hervey Bay were found to feed in the inner bay at high tide, hence favoring seagrass patches of low biomass but high starch and nitrogen concentration^37^. Such data on the biomass and nutritional contents of the seagrass patches where dugongs were tracked are not available in New Caledonia; they are needed to confirm whether dugongs adopt dynamic trade-offs in feeding strategies, whereby animals may favor foraging in areas of greatest nutrient return when the tides allow access to shallow seagrass meadows. When such areas are not accessible, dugongs may be restricted to using deeper habitats, in which they minimize foraging time and in turn energy expenditure, by feeding in targeted localized seagrass patches of high biomass.

This study reports an additional tracked dugong using the fore-reef shelf, a shallow (< 10 m) oceanic terrace located outside the lagoons adjacent to the barrier reef and devoid of seagrass. Furthermore, our reanalysis of the complete dugong tracking dataset in New Caledonia revealed that the tracked dugongs’ few excursions outside the lagoons occurred primarily at dawn, after spending the night in shallow waters inside the lagoon, presumably to feed. The timing and duration of these excursions was similar to those observed in Moreton Bay, eastern Australia^38^. The mechanisms underlying this rarely described behavior require further investigation. Indeed, the use of the fore-reef shelf by dugongs in New Caledonia was first reported by Garrigue et al.^43^ who observed animals forming relatively large herds (up to 80 individuals) in this habitat near Cap Goulvain (ecoregion 2; Fig. 1). Subsequent analysis of opportunistic aerial and underwater footages combined with local scale aerial surveys and analysis of water temperature suggested that a higher proportion of dugongs use the fore-reef shelf during the cool season when this habitat becomes warmer than the lagoon. The fore reef shelf could therefore be a place for dugongs to thermoregulate^65^. Similar inshore-offshore movements for thermoregulation were reported at the high latitudinal limit of the dugong range in Australia^32,38,66^. The first dugong tracking study conducted in New Caledonia then showed that the fore-reef shelf in ecoregion 2 was also used as a movement corridor^37^ (i.e. three tracked dugongs undertook transiting movements along the fore-reef shelf from Cap Goulvain to Bourail Bay located some 20 km southwards within ecoregion 2; individuals J, K, L in our study). Cleguer et al.^35^ suggested that the use of this oceanic habitat could partly be the result of the lagoon being very narrow in this region and that travelling outside the lagoon was likely safer for dugongs than swimming within the lagoonal shallow reticulated reefs, where predation from tiger sharks may occur ^16,43,67^. Interestingly, in this study we observed for the first time a dugong (individual N) using the fore-reef shelf in ecoregion 3, where the lagoon is relatively larger and deeper than in ecoregion 2. While over the fore-reef shelf, this dugong displayed variable residency times oscillating between transit like and ARS behavior. This new information suggest there may be more than lagoon size and depth to explain this unique use of the fore-reef shelf by dugongs and that this habitat may provide various advantages that require further investigation.

## Conclusion

Our knowledge of dugong movements in complex coral reef ecosystems has progressed beyond the simple exploration of individual movements to assess dynamic habitat use patterns in relation to the environment. Our study shows that integrating data from multiple source of observations (here telemetry tracking and systematic population aerial surveys) can enhance habitat use models. This study illustrates the value of designing scientific surveys and conservation measures at an ecoregional scale, particularly when working in complex ecosystems composed of diverse habitats. In such study areas, standardized approaches to identify ecoregions could be applied to ensure an adequate choice in tagging locations that reflect the entire diversity of wildlife habitat use patterns. The objective empirical identification of ecoregions to describe geographical adaptations in dugong habitat use provided ecologically meaningful knowledge that should inspire the design of future research on highly mobile wildlife species.

## Methods

### Ecoregions

#### Environmental data

Several static and dynamic environmental datasets were acquired to describe environmental conditions over the western coast of New Caledonia. Bathymetric charts at 100 m resolution were obtained from the MNT IRD-Shom^68^. Coastlines and coral reef contours were obtained from the Millenium Coral Reef Mapping Project^69,70^. Seagrass patches were obtained from a nation-wide seagrass mapping dataset derived from satellite imagery and in situ records in relatively shallow waters < 5 m (product L0ST including dense/medium/shallow envelop shapefiles^64^). This product included both confirmed and putative seagrass patches, to remain consistent with previous dugong habitat studies conducted in New Caledonia^35^.

Environmental variables were derived from these datasets over a grid of 500 m resolution projected in a UTM coordinate system and covering the study area. In each grid cell, the median depth (in m) and median seabed slope (in degrees) were calculated from bathymetry using the *raster* R package (version 3.0-12) and log-transformed. In addition, the euclidean distance (km) to the coast was calculated (excluding islets < 1 km^2^), as well as the euclidean distance to the barrier reef that separates the lagoon from the open ocean (i.e. distance to barrier reef) and the distance to the closest patch of intermediate shallow coral reef (i.e. distance to intermediate reef patches, excluding fringing and barrier reefs). Distance to barrier reef was modified to present negative distances when moving outwards from the barrier reef and positive distances when moving inwards. Distance to shallow seagrass patches (in km) and shallow seagrass coverage (% of × 500 m grid cells) were also calculated.

#### Categorizing ecoregions

The lagoon of the western coast of New Caledonia was divided into 14 slices of 20 km wide, angled at 45° with the coastline. The physical characteristics of the lagoon habitat were described within each slice based on nine environmental variables: the mean and standard deviation of distance to intermediate reef patches, the mean and standard deviation of slope, the mean and standard deviation of depth, the mean latitude, the total surface area of the slice (approximating the lagoon width) and the level of opening in the barrier reef. This last parameter was calculated by summing the width of all passes found in the barrier reef inside a slice. A hierarchical clustering using Ward's minimum variance method was applied to clusterize the 14 slices into a smaller number of ecoregions based on the physical characteristics of their habitats. In the clustering process, ecoregions were not constrained to form a spatially continuous assemblage of slices. Clustering was conducted for three, four or five ecoregions, and silhouette plots were used to select the best clustering^71^.

### Individual movements and residence time

#### Dugong tagging data

Sixteen dugongs were tagged over four sites along the western coast of New Caledonia: Koné, Cap Goulvain, Ouano, and Nouméa (Fig. 1). They were captured using the rodeo technique ^72,73^ and equipped with TMT-462-3 GPS/Argos transmitters (Telonics), hereafter referred to as GPS-satellite tags, in 2012, 2013 and 2019 (Table 2) as part of two independent studies. The first one documented the dugongs’ movement extent, heterogeneity and core areas of use in coral reef lagoon ecosystems ^35^ and the other investigated the use of a mining port and its vicinity by tagged animals ^74^. Details of the capture process, animal sexing and measurements can be found in those publications. All permits required to capture and satellite-track dugongs were obtained from the James Cook University Animal Ethics Committee (A1735 and A1936), Murdoch University (R3169/19) and the North (60912155-2013/JJC and 609011-52/2019/DE/JJC) and South (3157-2012/ARR/DENV) Provinces of New Caledonia. Captured dugongs were handled in strict accordance with local and international regulations using expert, veterinary advice (Mark Flint pers. comm.). The tags employed fast acquisition GPS tracking technology (Quick Fix Pseudoranging technology or QFP) developed for marine mammals that surface only briefly. The QFP technology obtains locations to an accuracy of within 10 m (www.telonics.com).

#### Track filtering and interpolation

GPS locations were filtered to remove invalid locations of uncertain QFP and unresolved QFP and locations implying unrealistically rapid movements (speed > 10 km/h^30^). The error on all GPS positions was set to 10 m. When a track was interrupted for more than 10 h, the track was considered to be constituted by several segments, which were subsequently modeled separately. Track segments were projected in a UTM coordinate system and were interpolated hourly with a Continuous-time Correlated Random Walk (CRW) model using the *crawl* R package (version 2.2.3 ^75^). CRW models movement as a velocity process, characterized by two parameters: β, the velocity autocorrelation, and σ, the velocity variation. Using these models, the animal’s position can subsequently be predicted at any time, from the start to the end of the original track. The β parameter was constrained between [− 3, 4] bounds and was optimized using a Normal distribution prior with mean 0.5 and standard deviation 2. The σ parameter was left unconstrained and was optimized with the L-BFGS-B method. Before and after applying the CRW model, the GPS locations erroneously located on land were shifted to be relocated into the closest waters using a custom algorithm (see Supplementary Code S4). Contrary to the common practice of removing such locations, this relocation algorithm allowed to conserve as many locations as possible to avoid creating gaps within tracks.

#### Residence time

Residence time was calculated along the crawl-interpolated tracks to assess movement type^76^. Residence time is the total amount of time spent, both backward and forward, within a virtual circle (of radius ρ) centered on a given location, provided the animal did not move out of the circle for more than a time threshold (τ). Residence time therefore provides an integrative measure of space use and may reveal differences between transit-like movement and ARS when animals slow down and/or display more sinuous paths as a result of a spatially-restricted activity (e.g., resting, feeding, or interacting with conspecifics). ARS behavior is scale-dependent, a pattern that can be tested using varying radii ρ in the residence calculation. Here, residence time was calculated in a radius ρ of 1, 2, 3 or 4 km (with a time threshold τ of 2 h) for each tagged individual. The log-transformed variance of the residence time values was calculated for each individual in order to determine the best study scale. The 1 km radius was found to maximize the variance of residence time for the majority of individuals and was selected for further analysis. Hourly residence time series estimated for each tagged dugong were subsequently smoothed with a moving average of order 4 with the *forecast* R package (version 8.12^77^), in order to remove noisy values in the movement trend. Speed was also calculated between hourly locations (in km/h). Residence time and speed were not estimated over the first 3 h and the last 3 h of each tracking segment. Residence time was compared between males and females with a linear mixed effect model accounting for individual variability, using the *nlme* R package (version 3.1-144^78^) with a Maximum Likelihood method.

### Dynamics of habitat use

#### Lagoon habitat use

Intensity of use was spatially estimated over a grid of 500 m resolution covering the study area and extending up to 2.5 km off the barrier reef (the furthest distance offshore a dugong was observed). Intensity of use was calculated for each tagged dugong as the average residence time per grid cell weighted by the surface of the grid cell not covered by land. The maximum mean intensity of use across all dugongs was selected per grid cell to form the response variable to be modeled as a function of static environmental predictors. Prior to producing habitat use models, Pearson coefficients were computed between pairs of environmental predictors within each ecoregion to prevent collinearity. As a result, slope and distance to shallow seagrass patches were discarded from the list of predictors as they bared strong correlations (r > 0.7) in more than one ecoregion, with depth, distance to the coast or distance to intermediate reef patches. Five predictors were finally retained: depth, distance to intermediate reef patches, distance to the barrier reef, distance to the coast and shallow seagrass coverage. Intensity of use was modeled as a negative binomial variable in a Generalized Additive Model (GAM^79^), using the Restricted Maximum Likelihood Method in the *mgcv* R package (version 1.8-31^80^). The effect of environmental predictors was assessed with penalized thin plate regression splines separately within each ecoregion. Splines basis size was limited to five to prevent overfitting^81^. Partial dependence plots^82^ were produced for each significant environmental predictor/ecoregion combination.

#### Tidal and diel cycles

Tidal cycle data were acquired from in situ tide gauge measurements at the Numbo station in Nouméa, New Caledonia (REFMAR, data.shom.fr). Sea surface height was recorded every 20 min and processed with the *Tides* R package (version 2.0^83^). Records were categorized as low tide or high tide when sea surface height was respectively within 1 h before/after the peak minimum or maximum sea surface height. Diel cycle was approximated by dividing days into four phases: dawn (3–9 a.m), day (9 a.m–3 p.m), dusk (3–9 p.m) and night (9 p.m–3 a.m).

Linear mixed models were used to estimate the combined effect of the tidal and diel cycles on dugong residence time, and on the depth range dugongs occupied in each of the ecoregions previously identified. Individual-level random effects allowed the model’s intercept to vary among individuals found in each ecoregion. Individuals with less than 10 locations in an ecoregion were removed. Residence time was log-transformed to satisfy the assumption for normality of residuals while considering that the variance of the error term increases with an increase in response values^84^. Depth was binned into three categories, relevant to the general topography of the New Caledonia west coast lagoon: shallow [0, 2] m, medium [2, 10] m, and deep > 10 m. Models were fit with the *nlme* R package using a Maximum Likelihood method. Residuals were checked with standard diagnostic plots. Post-hoc tests on marginal means were run with Tukey corrections using the *emmeans* R package (version 1.4.7^85^). Marginal R^2^ and conditional R^2^ were reported to assess the proportion of variance explained respectively by the fixed factors only, and by the combined fixed and random factors. Finally, the timing of excursions to the fore-reef shelf was specifically considered to assess the effect of the dial cycle on outwards and inwards movements pass the barrier reef. χ^2^ goodness of fit tests (with simulated p-values based on 2000 replicates) were used to test whether outwards and inwards movements were randomly distributed across diel categories (dawn, day, dusk, night).

### Habitat use model cross-validation

In order to assess the ecological relevance and novel patterns revealed by our habitat use model, predicted intensity of use was compared with dugong densities estimated from aerial surveys conducted around the main island of New Caledonia between 2003 and 2012^7^. Intensity of use was predicted by our model over the gridded study area of resolution 500 m and transformed with a square root function. The highest 10% were considered as highly suitable habitat, while the lowest 10% were considered poor habitat. The comparison of these predicted values with relative dugong density estimated at a resolution of 1.6 km by Cleguer et al.^7^ revealed discrepancies between the two datasets: (1) areas predicted with highly suitable habitat but where density was classified as low (0 dugongs/km^2^) were reported as “newly predicted”, and (2) areas predicted with poor habitat but where density was classified as high (0.10–0.50 dugongs/km^2^) to very high (> 0.5 dugongs/km^2^) were reported as “omitted”. Such cross-validation was performed within the three depth ranges used for the analysis of tidal and diel effects (shallow [0, 2] m, medium [2, 10] m, deep [10, 60] m), plus a very deep water category (> 60 m) to better describe the deep waters located off the barrier reef. Linear relationships between predicted intensity of use and relative density were estimated with Pearson coefficients.

Data processing, analyses and mapping were conducted with the R software (version 3.6.3^86^).

## Supplementary Information

Supplementary Information.

## Data Availability

The data used in this manuscript and the shapefiles of key dugong habitats are available on request in the online repository DataSuds (https://dataverse.ird.fr/dataverse/dugong_habitat_use_nc).
