# Supplementary material for: Ecoregional and temporal dynamics of dugong habitat use in a complex coral reef lagoon ecosystem
Source: Sci Rep. 2022 Jan 11;12:552. doi: 10.1038/s41598-021-04412-3 (PMC8752826; doi:10.1038/s41598-021-04412-3)
Supplement: Supplementary file 1 — Supplementary Information. [file 41598_2021_4412_MOESM1_ESM.pdf]

Ecoregional and temporal dynamics of dugong habitat use in a complex coral reef lagoon ecosystem

Solène Derville [+*1,2,3] and Christophe Cleguer [+4, 5, 1], Claire Garrigue [1,2]

[1] UMR ENTROPIE (IRD-Université de La Réunion-CNRS-Laboratoire d'excellence LabEx-CORAIL), 98800, New Caledonia

[2] Opération Cétacés, Nouméa, 98802, New Caledonia

[3] Marine Mammal Institute, Oregon State University, 2030 SE Marine Science Dr., Newport, OR 97365, United States

[4] Centre for Sustainable Aquatic Ecosystems, Harry Butler Institute, Murdoch University, Murdoch 6150, Western Australia, Australia

[5] Centre for Tropical Water & Aquatic Ecosystem Research (TropWATER), James Cook University, Townsville 4811, Australia

*Corresponding author: Solène Derville s.derville@live.fr

[+]Solène Derville (ORCID 0000-0002-0380-7921) and Christophe Cleguer (ORCID 0000-0002-9026-336X) should be considered joint first author

SUPPLEMENTARY INFORMATION

Figure S1 – Ecoregion principal component analysis (PCA) and hierarchical clustering. PCA was applied to 10 environmental variables describing lagoon topography in each of the 14 slices covering the west coast lagoon of New Caledonia. Lagoon topography was described by the mean and standard deviation of distance to intermediate reef patches (‘disreef.mean’, ‘disreef.sd’), the mean and standard deviation of slope (‘logslope.mean’, ‘logslope.sd’), the mean and standard deviation of depth (‘logdepth.mean’, ‘logdepth.sd’), the mean latitude (‘lat’), the total surface area of the slice (‘area’) and the level of opening in the barrier reef (‘channel_width’). The contribution of environmental variables to the two primary PCA axes are represented in the upper left panel, as well as the distribution of the sample points within this space in the lower right panel. Ultimately, slices were categorized into three ecoregions (ecoregion 1 in blue, ecoregion 2 in red, ecoregion 3 in yellow, in the lower left panel where slices are numbered from 1 to 14) using a hierarchical clustering with Ward's minimum variance method. A dendrogram represents the clusters formed by the 14 slices (upper right panel).

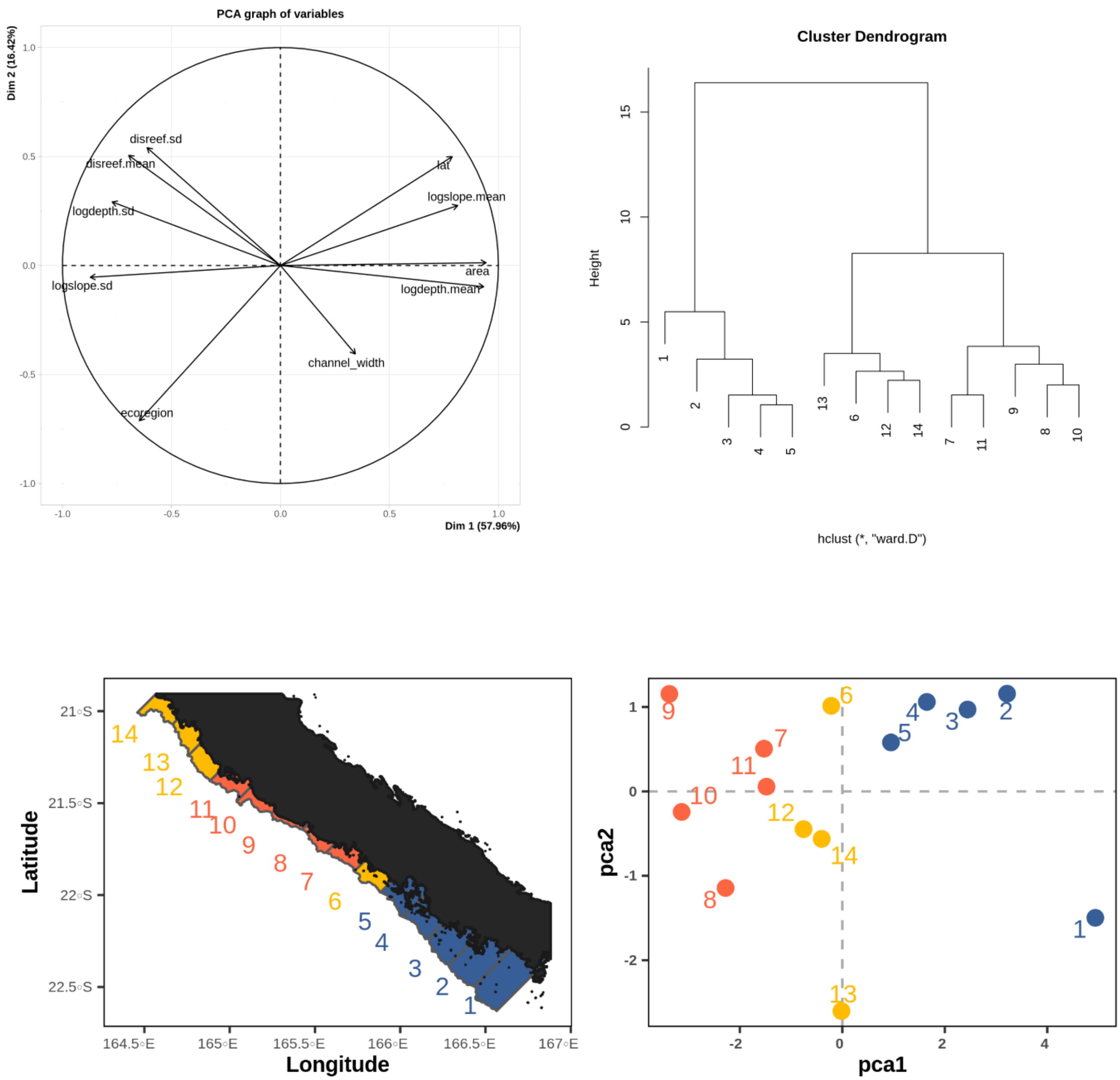

Figure S2 – Individual maps of residence time along dugong tracks. The color gradient representing residence time (RT, in hours) at each location is log scaled. The locations of tag deployments are shown with white diamond shapes. Tracked individuals are named from A to P, as referenced in Table 2. Land is shown in black and shallow reefs are shown in grey (shapefile source: Millenium Coral Reef Mapping Project[69,70]). The maps were created with R (version 3.6.3, www.r-project.org [86]).

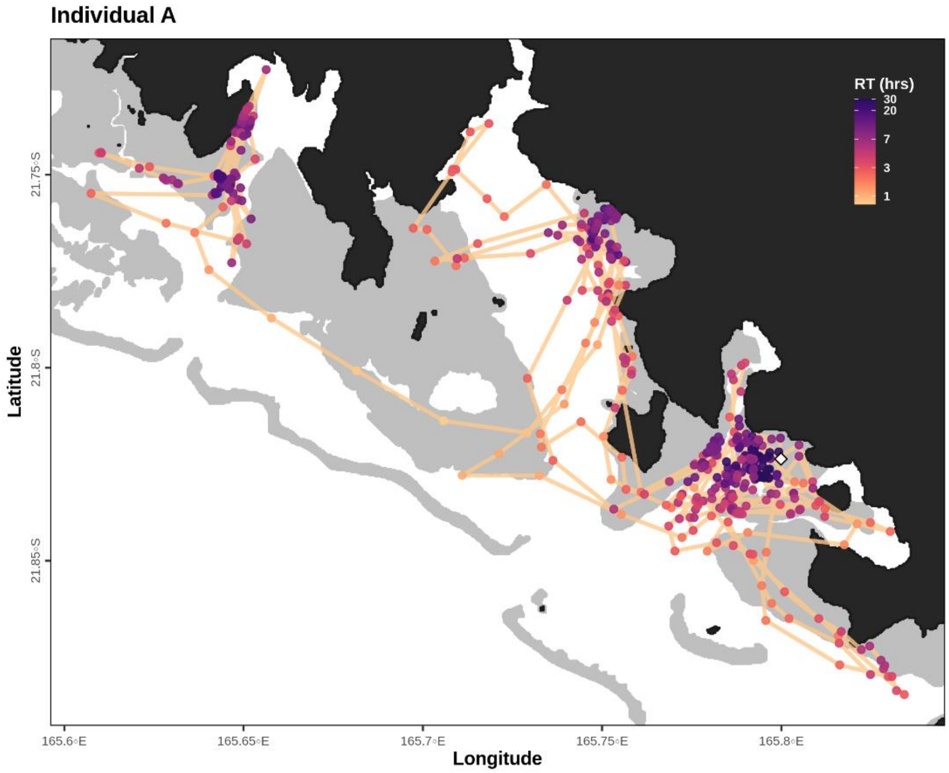

Individual B

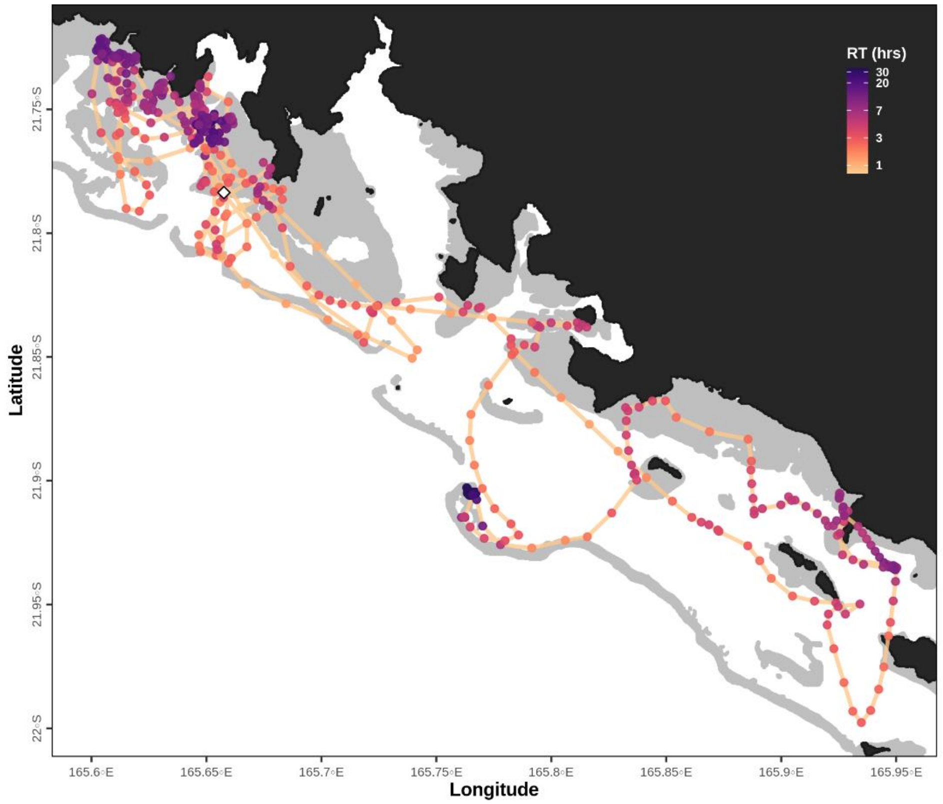

Individual C

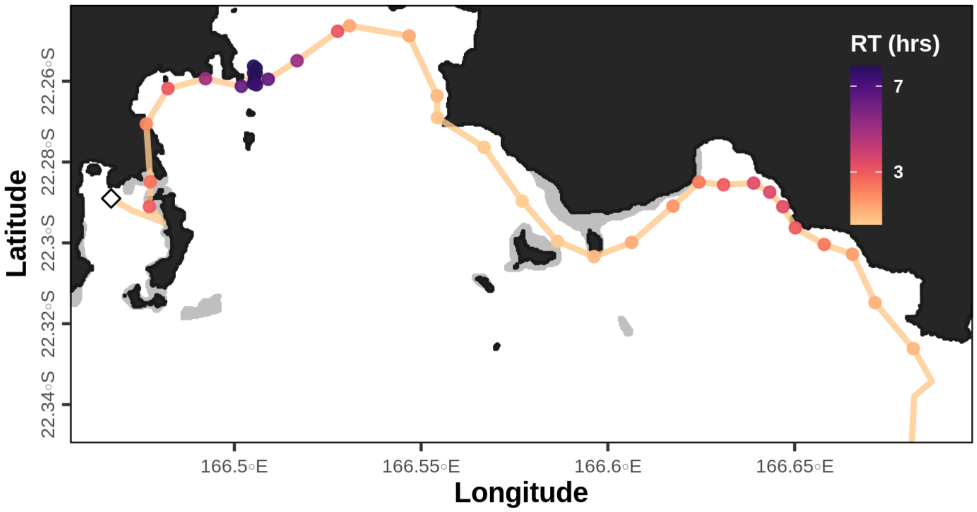

Individual D

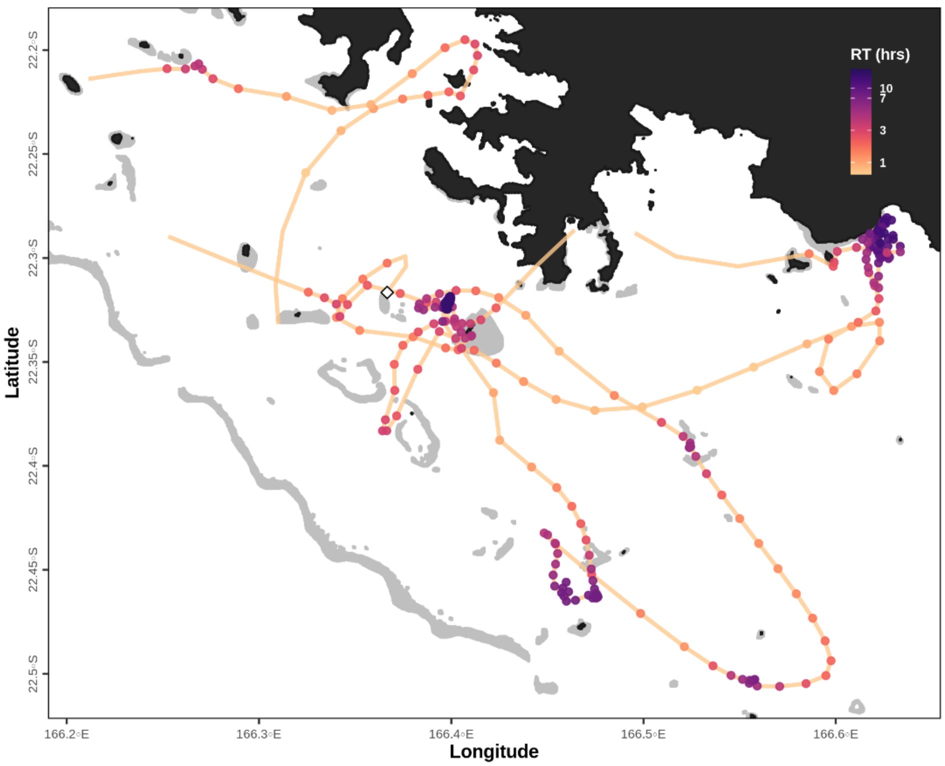

Individual E

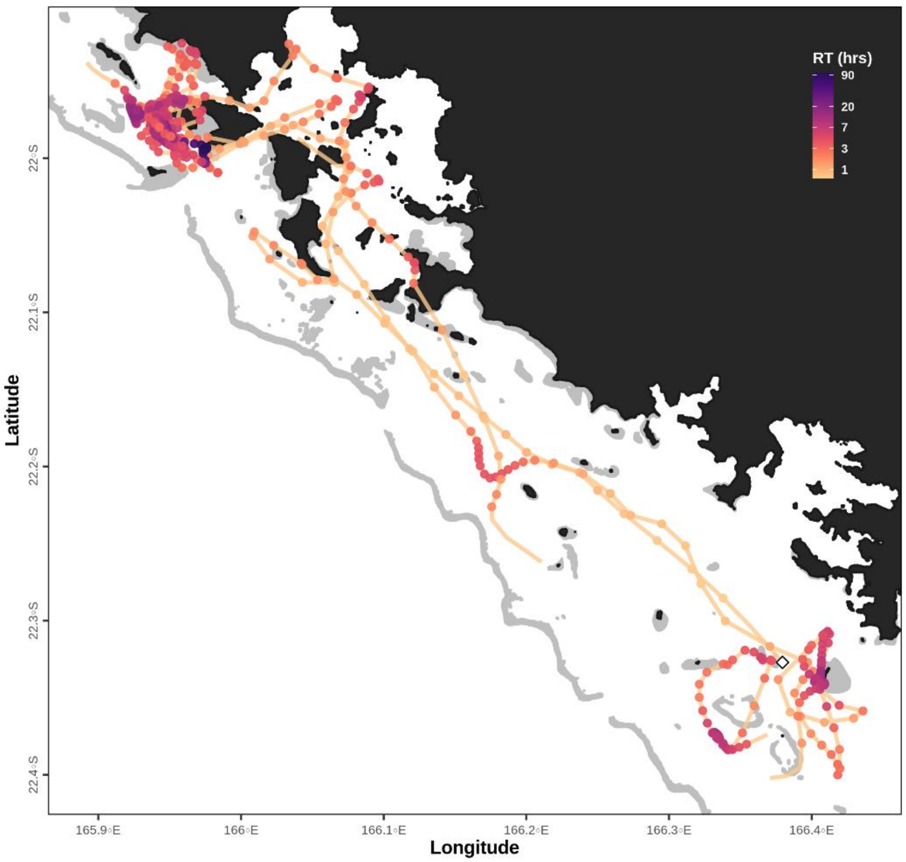

Individual F

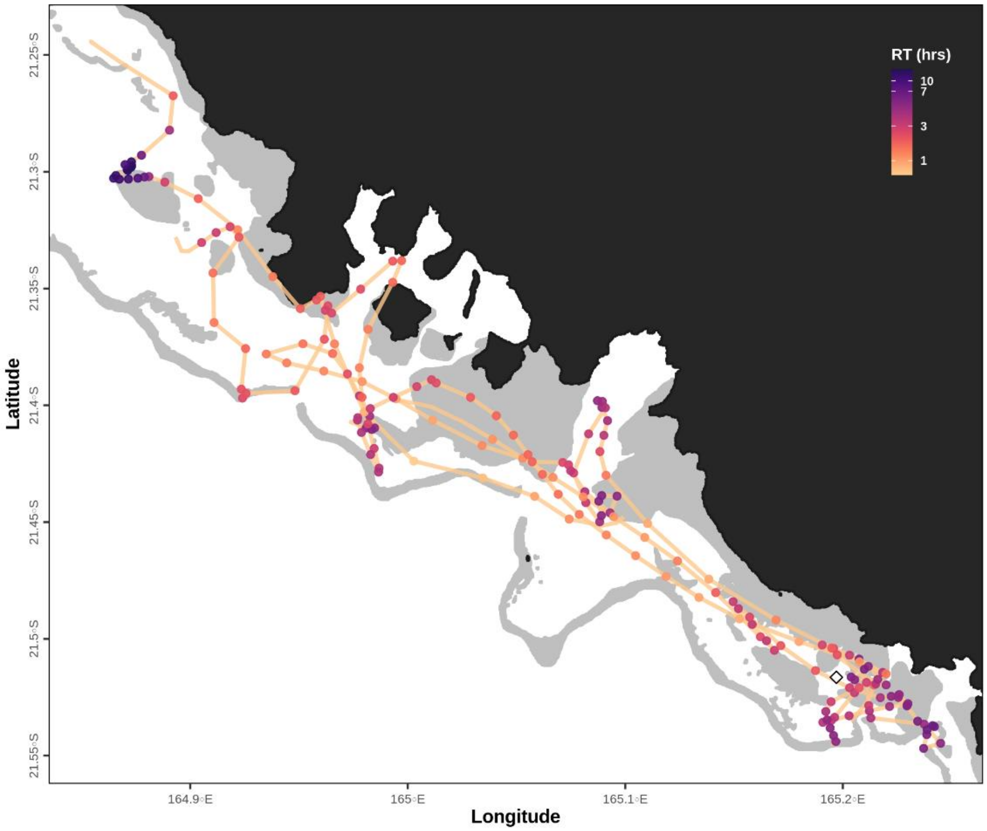

Individual G

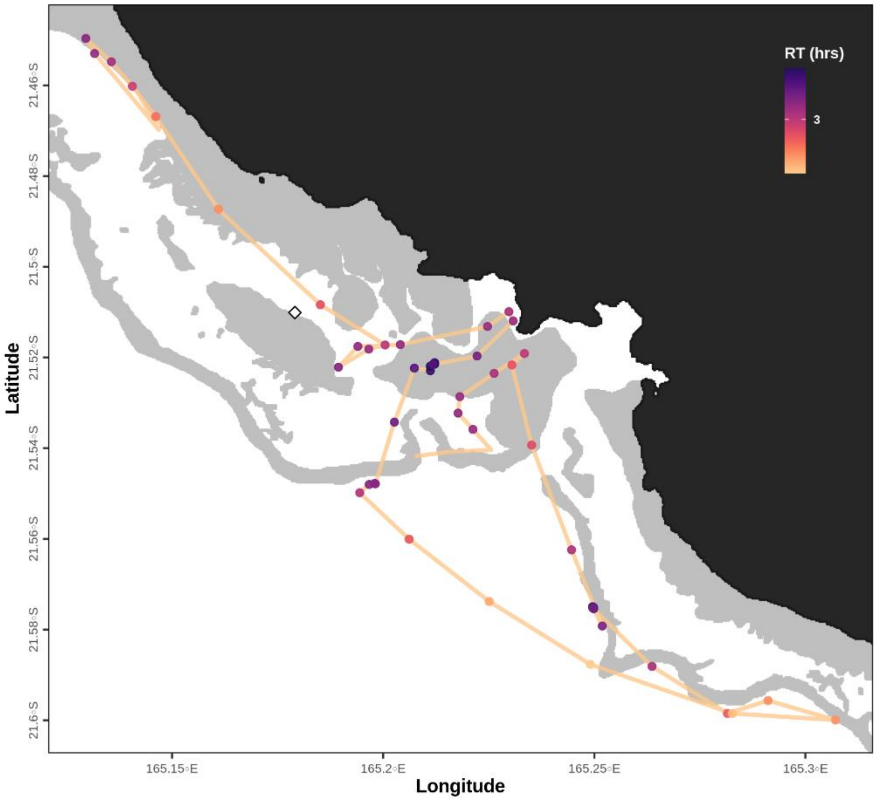

Individual H

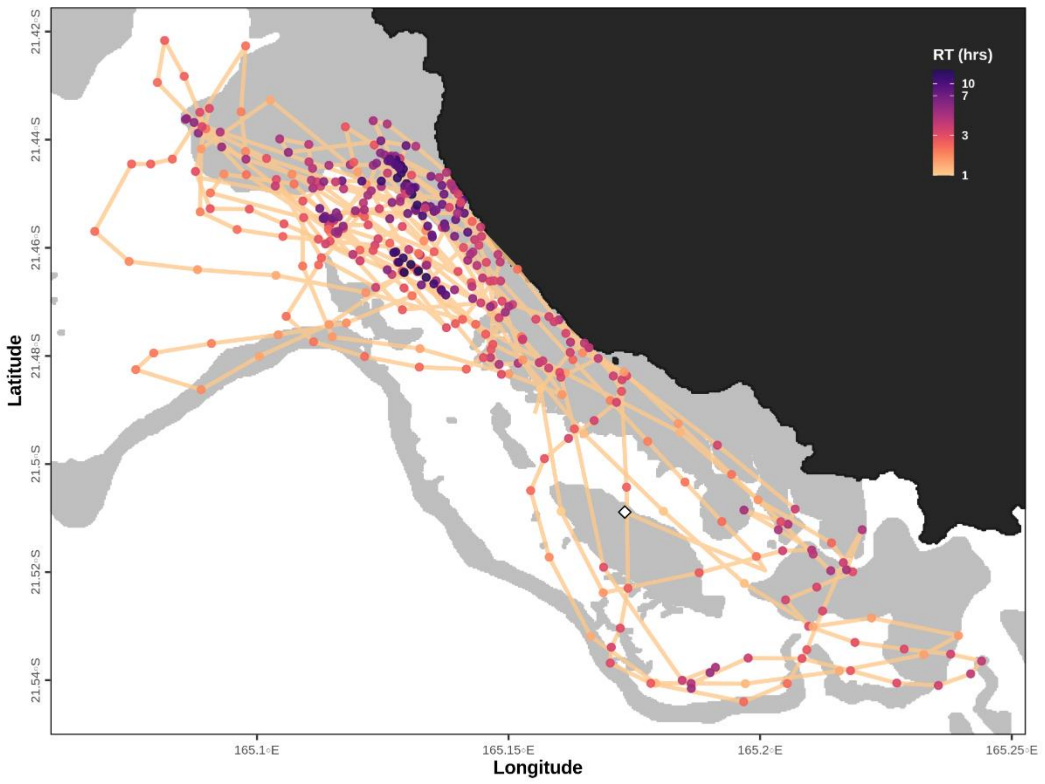

Individual I

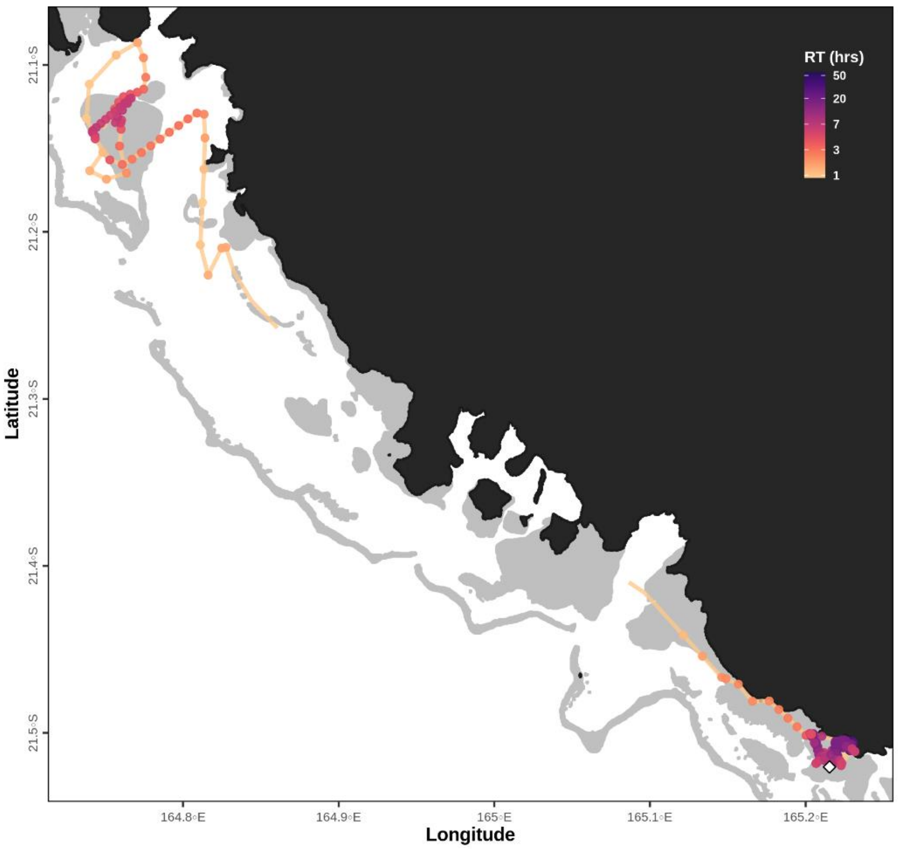

Individual J

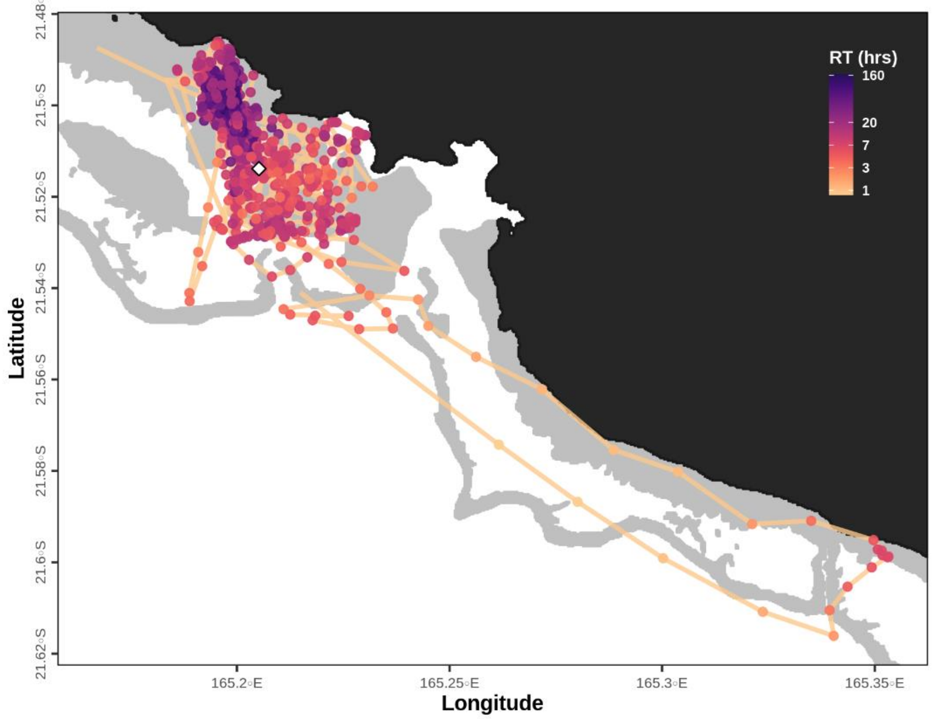

Individual K

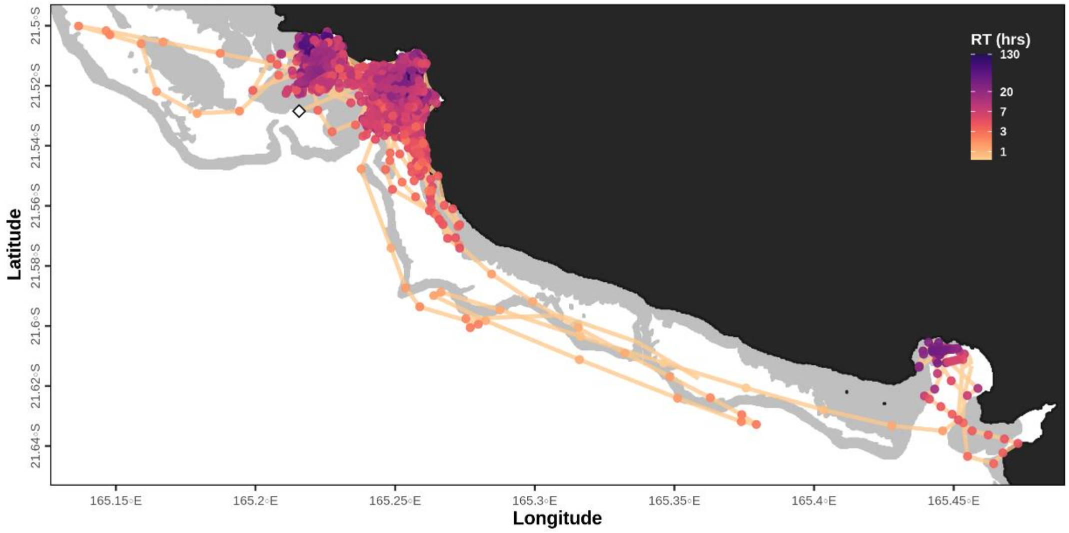

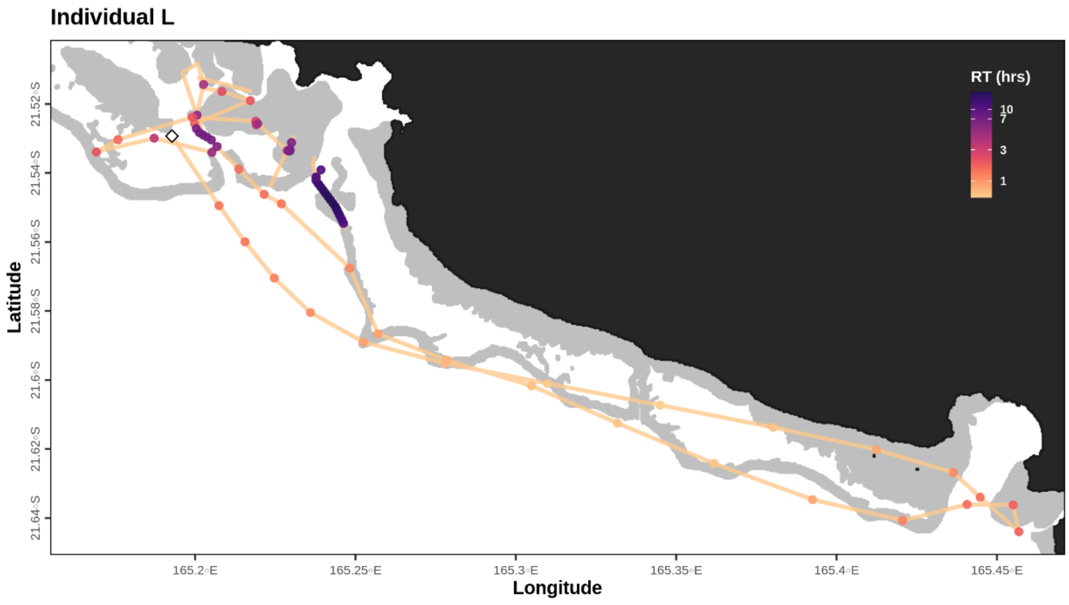

Individual L
RT (hrs)
10
7
3
1
Latitude
21.52◦S
21.54◦S
21.56◦S
21.58◦S
21.6◦S
21.62◦S
21.64◦S
165.2◦E
165.25◦E
165.3◦E
165.35◦E
165.4◦E
165.45◦E
Longitude

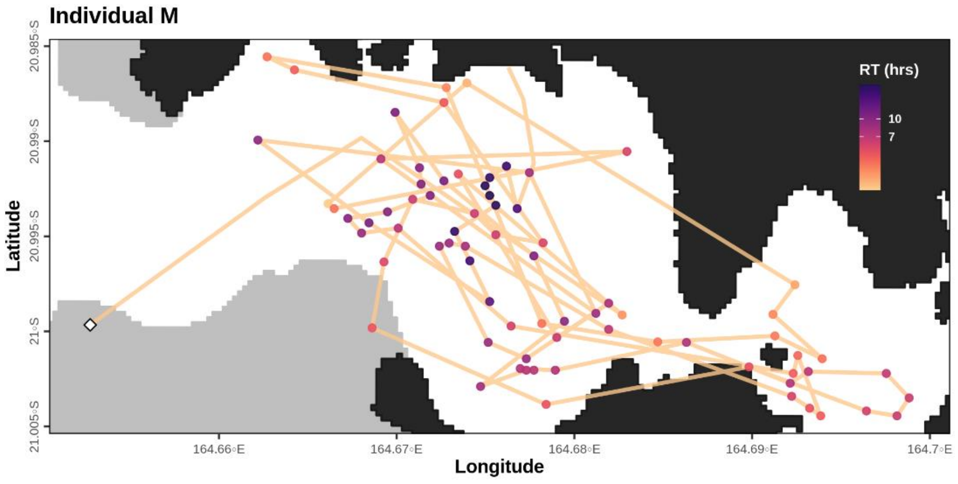

Individual M
RT (hrs)
10
7
Latitude
20.985◦S
20.99◦S
20.995◦S
21◦S
21.005◦S
164.66◦E
164.67◦E
164.68◦E
164.69◦E
164.7◦E
Longitude

Individual N

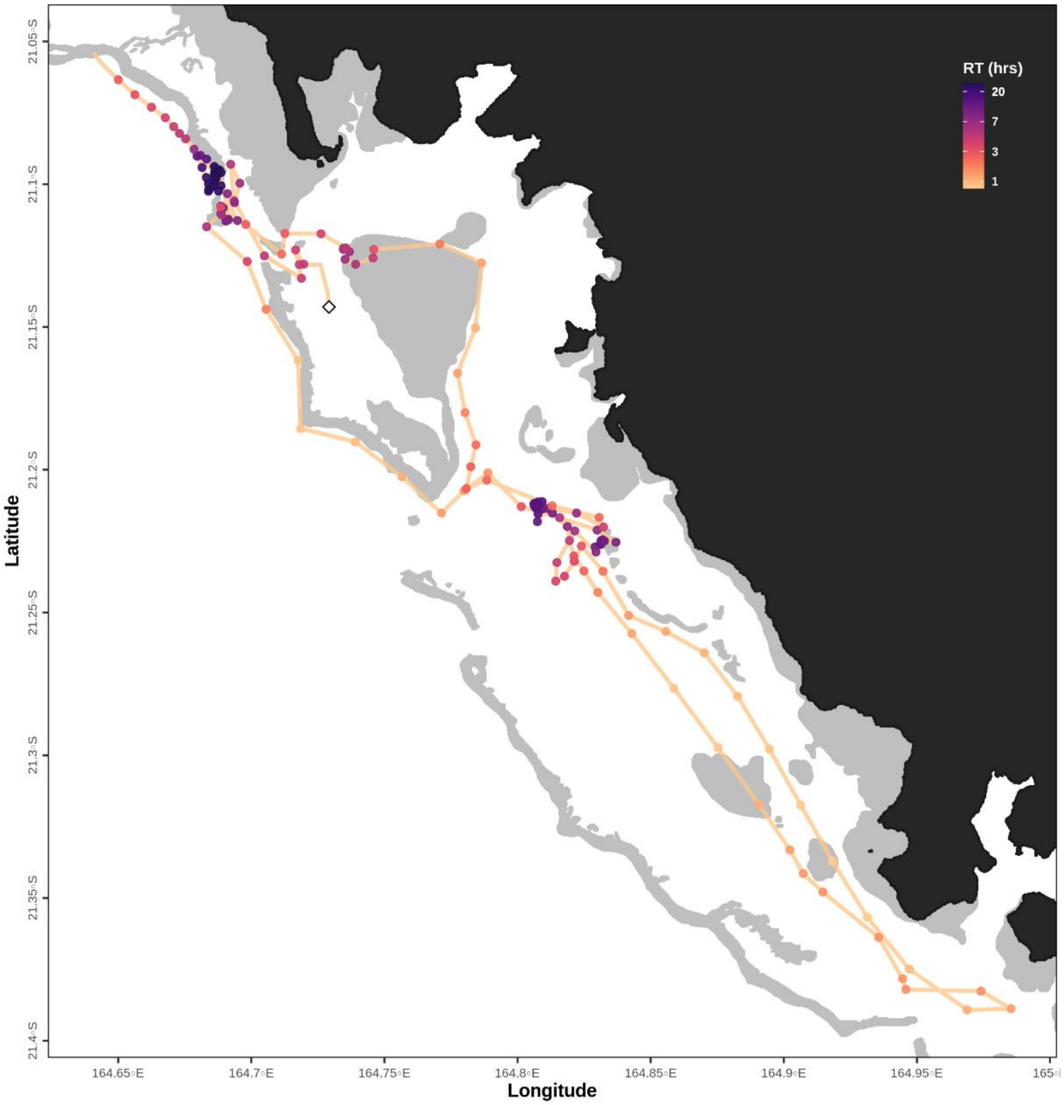

Individual O

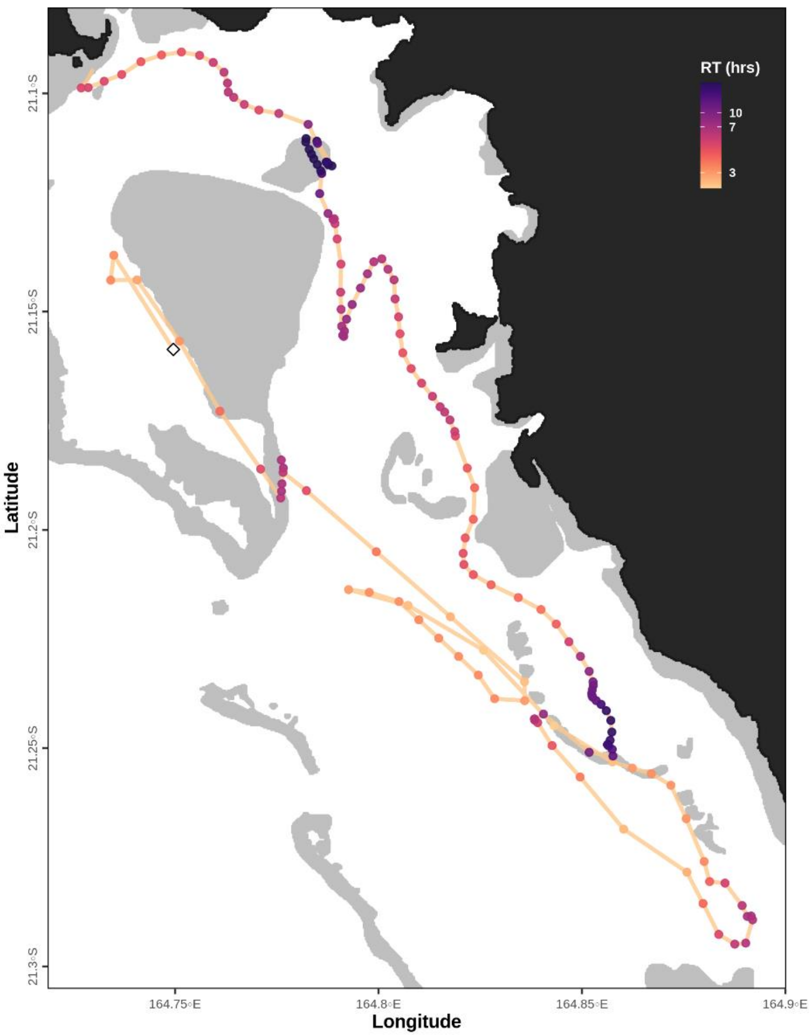

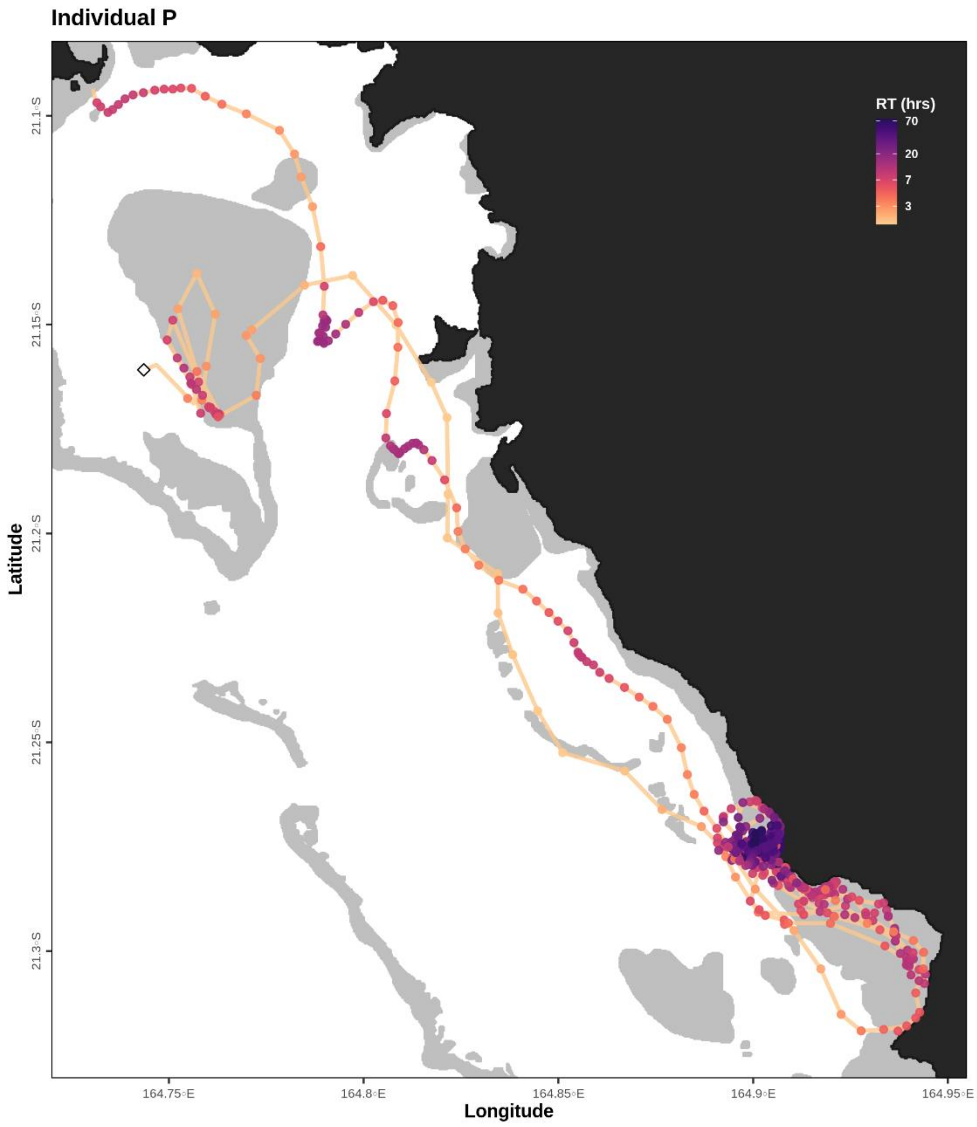
Individual P
RT (hrs)
70
20
7
3
21.1°S
21.15°S
21.2°S
21.25°S
21.3°S
Latitude
164.75°E
164.8°E
164.85°E
164.9°E
164.95°E
Longitude

Table S3 – Summary of dugong excursions outside the barrier reef. The local time (NC time zone), tidal and dial phase during outwards (coming out of the lagoon) and inwards movements (coming back inside the lagoon) are reported, along with the duration of the excursion and the mean residence time (in hours) calculated over locations outside the lagoon. Tracked individuals are named from A to P, as referenced in Table 2.

Animal	Ecoregion	Time outwards	Tidal outwards	Dial outwards	Time inwards	Tide inwards	Dial inwards	duration	mean RT
N	3	11/10/2019 06:00:00	High	Dawn	11/10/2019 13:00:00	Low	Day	07:00	2.0
N	3	15/10/2019 13:00:00	Ebb	Day	17/10/2019 00:00:00	Ebb	Night	11:00	19.0
L	2	03/10/2013 12:00:00	Low	Day	03/10/2013 19:00:00	High	Dusk	07:00	1.0
L	2	04/10/2013 11:00:00	Ebb	Day	04/10/2013 13:00:00	Low	Dawn	02:00	1.4
K	2	28/01/2014 06:00:00	High	Dawn	28/01/2014 15:00:00	Flow	Dusk	09:00	1.5
K	2	02/02/2014 06:00:00	Flow	Dawn	02/02/2014 13:00:00	Ebb	Day	07:00	1.8
J	2	05/10/2013 06:00:00	Flow	Dawn	05/10/2013 16:00:00	Flow	Dusk	10:00	3.9
J	2	06/10/2013 08:00:00	High	Dawn	06/10/2013 15:00:00	Low	Dusk	07:00	1.2
G	2	06/10/2013 23:00:00	Ebb	Night	07/10/2013 18:00:00	Flow	Dusk	19:00	3.2
B	2	09/03/2012 08:00:00	High	Dawn	09/03/2012 10:00:00	Ebb	Day	02:00	1.3

Code S4 – Custom R code for shifting GPS locations on land

```r
library(sf) ; library(plyr)
# Function created to slightly shift the points of a track that fall on land (due to GPS accuracy or to shoreline imprecision)
#### INPUT is the dataframe with the track positions
# coord_names is the names of the columns with the projected coordinates
# land_shape is the sf spatial object containing the land polygons
# crs is the EPSG code for the desired projected coordinate system. Here 32758 was used.

#### OUTPUT is a list with three elements: 1- the dataframe with corrected points,
# 2- the points that were on land
# 3- the corrected points shifted to be in the water at least 100 m from shore
Fun_ShiftOnLand <- function(df, coords_names, land_shape, crs){
  # convert to spatial object sf
  fit_sf <- st_as_sf(df, coords = coords_names, crs = crs)
  fit_sf$ref_point <- row.names(df)
  points_on_land <- st_intersects(fit_sf, land_shape, sparse = T)
  points_on_land_df <- fit_sf[apply(points_on_land, 1, any), ]

  ###############
  # move these points to the closest positions not on land
  points_jittered <- list()
  for (i in 1:100){
    p <- st_jitter(points_on_land_df, amount = 2000, factor = 1) # generate points in a 2 km buffer
    p <- p[!apply(st_intersects(p, land_shape), 1, any),] # remove those on land
    points_jittered <- c(points_jittered, list(p))
  }
  points_jittered <- ldply(points_jittered, rbind)
  points_jittered <- st_as_sf(points_jittered)
  # only consider large lands and remove islets < 1 km2
  land_masses_utm <- land_shape[as.numeric(st_area(land_shape)) > 1e6, ]
  # calculate distance to land (in meters)
  points_jittered$dist_land <- as.numeric(st_distance(points_jittered, st_combine(land_masses_utm), which = "Euclidean")[,1])
  # remove points that are less than 100 m from shore
  points_jittered <- points_jittered[points_jittered$dist_land > 100, ]

  points_corrected <- ddply(points_jittered, ~ref_point, function(d){ # per point that needed moving
    d$dist_point <- as.numeric(st_distance(st_as_sf(d), points_on_land_df[points_on_land_df$ref_point == d$ref_point[1], ]))
    d$dist_mix <- rowSums(d[c("dist_land", "dist_point")])
    dd <- d[d$dist_mix == min(d$dist_mix), ] # select the point closest to shore and closest to its reference point
    return(dd)
  })
  points_corrected <- st_as_sf(points_corrected)
  # remove columns
  points_corrected$dist_land <- NULL
  points_corrected$dist_point <- NULL
  points_corrected$dist_mix <- NULL

  # remove the points that were on land
  fit_sf <- fit_sf[!apply(points_on_land, 1, any), ]
  # replace them by the jittered points
  fit_sf <- rbind(fit_sf, points_corrected)
  # convert back to dataframe
  fit_sf[coords_names] <- st_coordinates(fit_sf)
  fit_sf$geometry <- NULL
  fit_sf$ref_point <- NULL

  list_out <- list(updated_dataframe = fit_sf,
                   points_on_land = points_on_land_df,
                   points_corrected = points_corrected)
  return(list_out)
}
```
